# A systematic review of the applications of Expert Systems (ES) and machine learning (ML) in clinical urology

**DOI:** 10.1186/s12911-021-01585-9

**Published:** 2021-07-22

**Authors:** Hesham Salem, Daniele Soria, Jonathan N. Lund, Amir Awwad

**Affiliations:** 1 Urological Department, NIHR Nottingham Biomedical Research Centre, School of Medicine, University of Nottingham, Nottingham, NG72UH UK; 2 University Hospitals of Derby and Burton NHS Foundation Trust, Royal Derby Hospital, University of Nottingham, Derby, DE22 3DT UK; 3 School of Computer Science and Engineering, University of Westminster, London, W1W 6UW UK; 4 NIHR Nottingham Biomedical Research Centre, Sir Peter Mansfield Imaging Centre, School of Medicine, University of Nottingham, Nottingham, NG72UH UK; 5 Department of Medical Imaging, London Health Sciences Centre, University of Hospital, Schulich School of Medicine and Dentistry, Western University, London, ON Canada

## Abstract

**Background:**

Testing a hypothesis for ‘factors-outcome effect’ is a common quest, but standard statistical regression analysis tools are rendered ineffective by data contaminated with too many noisy variables. Expert Systems (ES) can provide an alternative methodology in analysing data to identify variables with the highest correlation to the outcome. By applying their effective machine learning (ML) abilities, significant research time and costs can be saved. The study aims to systematically review the applications of ES in urological research and their methodological models for effective multi-variate analysis. Their domains, development and validity will be identified.

**Methods:**

The PRISMA methodology was applied to formulate an effective method for data gathering and analysis. This study search included seven most relevant information sources: WEB OF SCIENCE, EMBASE, BIOSIS CITATION INDEX, SCOPUS, PUBMED, Google Scholar and MEDLINE. Eligible articles were included if they applied one of the known ML models for a clear urological research question involving multivariate analysis. Only articles with pertinent research methods in ES models were included. The analysed data included the system model, applications, input/output variables, target user, validation, and outcomes. Both ML models and the variable analysis were comparatively reported for each system.

**Results:**

The search identified n = 1087 articles from all databases and n = 712 were eligible for examination against inclusion criteria. A total of 168 systems were finally included and systematically analysed demonstrating a recent increase in uptake of ES in academic urology in particular artificial neural networks with 31 systems. Most of the systems were applied in urological oncology (prostate cancer = 15, bladder cancer = 13) where diagnostic, prognostic and survival predictor markers were investigated. Due to the heterogeneity of models and their statistical tests, a meta-analysis was not feasible.

**Conclusion:**

ES utility offers an effective ML potential and their applications in research have demonstrated a valid model for multi-variate analysis. The complexity of their development can challenge their uptake in urological clinics whilst the limitation of the statistical tools in this domain has created a gap for further research studies. Integration of computer scientists in academic units has promoted the use of ES in clinical urological research.

## Introduction

In the 1950’s J McCarthy in Stanford University and A Turing in Cambridge University proposed the concept of machine simulation of human learning and intelligence [1, 2]. Being keen mathematicians, they advanced the basic mathematical logic into programming languages enabling machines to perform more complex functions. E Shortliffe advanced those systems to develop MYCIN, which successfully simulated the reasoning of a human microbiologist in diagnosing and treating patients with microbial infection [3]. Their model introduced Expert Systems (ES) to the scientific literature and a ten year review by Liao et al. demonstrated their wide prevalence in the industrial fields with immense applications including health care [4]. In contrast to Liao’s review, other studies questioned their real time implementation in health care and suggested a lack of their uptake and integration in the health care systems [5]. This is despite evidence from systematic reviews demonstrating the positive impact of computer aid systems on patients’ outcome and health care [6, 7].

This study aimed to systematically review published ES in urological health care with a primary aim to demonstrate their availability, progression, testing and applications. The secondary aim was to evaluate their development life cycle against standards suggested by O’Keefe and Benbasat in their review articles on ES development [8, 9]. The later would evaluate the gap between their development and implementation in health care.

## Methods

**Fig. 1:**
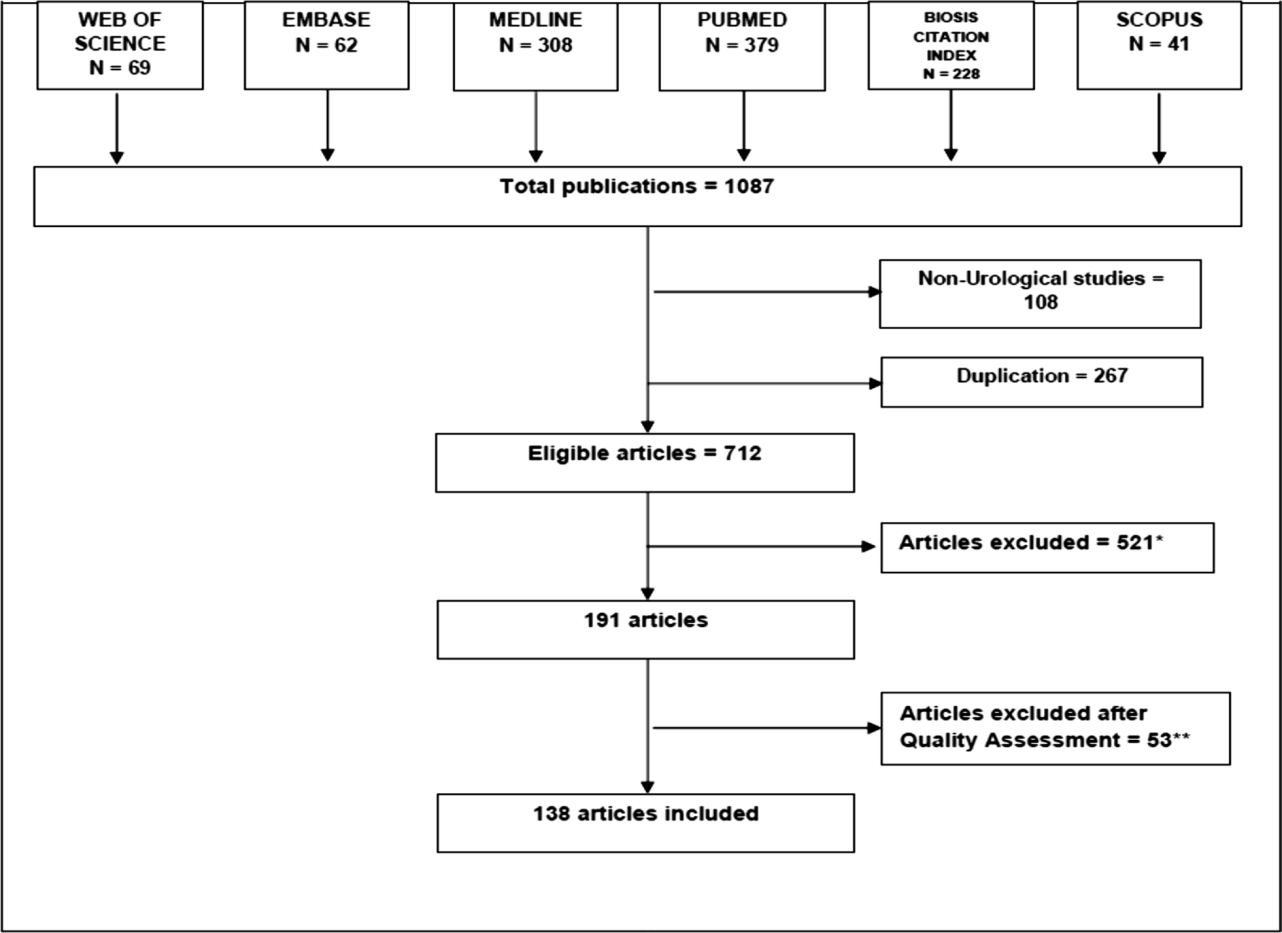
PRISMA flow chart for the systematic review of articles included in the review of expert systems in urology

The study methodology followed the recommendations outlined in the Preferred Reporting Items for Systematic Reviews and Meta-Analyses (PRISMA) statement (Fig. 1). No ethical approval was required because the type of the study waives this requirement.

### Search

Information sources including WEB OF SCIENCE, EMBASE, BIOSIS CITATION INDEX, SCOPUS, PUBMED, Google Scholar and MEDLINE were searched using key words in (Table 1). Articles published between 1960 and 2016 were considered and examined against the inclusion criteria. While tailoring the conducted search for each literature database, the key words were combined by “OR” in each domain, then domains were combined by “AND”.

**Table 1:** Keywords used for literature search

#1	**TOPIC:** ("expert system*") OR **TOPIC:** ("decision support") OR **TOPIC:** ("artificial intelligence") OR **TOPIC:** ("rule based") OR **TOPIC:** ("knowledge base* system*") OR **TOPIC:** ("neural network") OR **TOPIC:** ("fuzzy") DocType = All document types; Language = All languages;
#2	**TOPIC:** (urology) DocType = All document types; Language = All languages;
#3	#1 AND #2 DocType = All document types; Language = All languages;

### Eligibility criteria

For the primary aim, data search was conducted to yield the collected results then analyse them according to pre-planned eligibility criteria based on the system model, year of production, type and outcome of its validation, functional domain application, variables for input and output, target user and domain. This selection criteria were designed with an objective to identify expert system studies and demonstrate their prevalence, testing, and applications in clinical urology. Only articles and studies written in English were included.

Further qualitative analysis was required to meet the study secondary aim. For this, further data was gathered on credibility (user perception on the system), evaluation (system usability), validation (building the right system) and verification (building the system right) then compare against the standards reported in [8, 9].

### Data filtering

The resultant reference list of each included article was checked to identify a potentially eligible item that had not been retrieved by the initial search. All retrieved articles were collated in a final reference list on a management software (Endnote, X8), then duplicate studies were removed from the list.

Upon including more than one hundred articles, the rest of the eligible articles were meticulously compared to the ones included, then excluded based on demonstrating clear similarity. This was applied to avoid expanding the size of the data without adding to the study analysis.

## Results

**Fig. 2:**
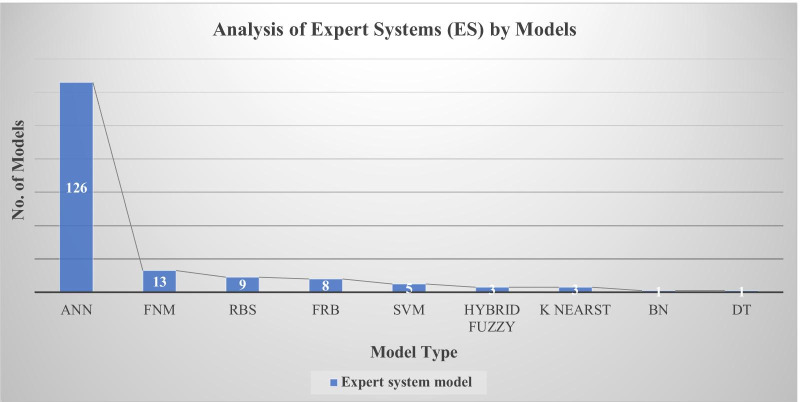
Analysis of Expert Systems (ES) by models (n = 169). ANN was the most common but other systems were applied on different domain as fuzzy neural model (FNM), rule-based system (RBS), fuzzy rule based (FRB), support vector machine (SVT), Bayesian network (BN) and decision trees (DT)

ANN was the commonest model to be applied in Urological ES (Fig. 2). The rest of the models demonstrated diversity which is consistent with other published industrial systems [4].

**Fig. 3:**
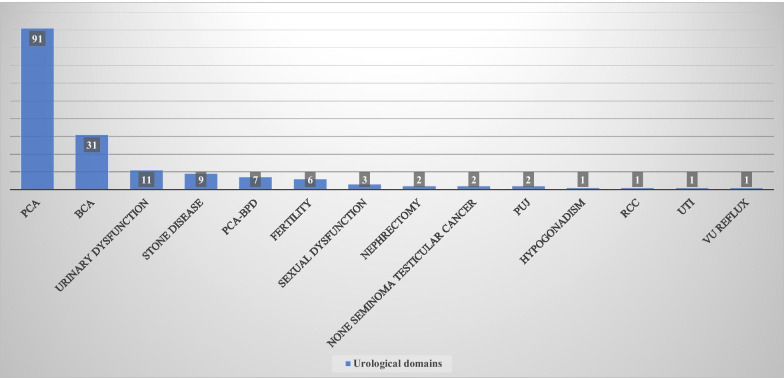
Urological domains (n = 168) applied by Expert Systems (ES). Prostate cancer (CaP) was the commonest domain followed by bladder cancer (Bca) then other diseases as benign prostatic disease (BPD), pelvi ureteric junction obstruction (PUJ), urinary tract infection (UTI), renal cell cancer (RCC), vesico ureteric reflux (VU reflux)

Prostate cancer was the commonest domain for urological ES with most of the system focusing on cancer diagnosis. These systems were applied to various domains (Fig. 3), and they were further stratified and analysed according to their core functional application as outlines in the methodology.

### Quantitative analysis

#### Decision support systems

The main objective of ES in this domain was to facilitate the clinical decision making by identifying key elements from patients clinical and laboratory examinations then refine a theoretical diagnostic or treatment strategy [10]. They can guide the expert to find the right answer [11] or take over the decision making to support the none expert as [12] or even replace both to interact with the patient directly [13].

They have supported various aspects of urological decision making such as diagnosis, investigations analysis, radiotherapy dose calculation, the delivery of behavioural treatment and therapeutic dialogues.

##### Domains

*Urinary dysfunction* (U Dys) was the commonest domain to be covered in the decision support system application (n = 9), which could be further categorised into U Dys diagnostic, investigation analysis and therapeutic systems. They have demonstrated a range of methodologies, validation, and target users (Table 2) applicable to Decision support systems in Urological domain. For instance, Keles et al. [14] designed an ES to support junior nurses in diagnosing urinary elimination dysfunction in a selected group of patients while [15, 16] systems were able to support any medical user to diagnose urinary incontinence with an accuracy reaching higher than 90%. The target user of most of these systems were predominantly medical health care workers including both experts and none experts, with exception of [13, 17] which can be directly used by patients to receive an assessment of their urinary elimination dysfunction followed by a tailored treatment plan.

**Table 2:** Decision support systems in urological domain

Article	Mdl	Dom	Subdomain	Variables	Output	Knowledge acquisition	Validation method	Target user
[18]	RBR	U Dys	Incontinence in long-term care facilities	Disease related questions	Recommendations	Experts	Comparison to blinded experts and pilot RCT	Non-expert nurses
[15]	RBR	U Dys	U incont treatment	Incontinence symptoms	Behavioural treatment	Agency guidelines	RCT (60) reliability and validity by experts	Patients
[19]	RBR	U Dys	U incont treatment	19 evaluation questionnaires	Individualised health information	An expert and patients’ feedback	No validation	Patients
[20]	RBR	U Dys	U incont	MH, incontinence symptoms, previous incidents and medication history	U incont treatment	Multiple experts, patients record and literature	Evaluation by experts, 95 retrospective data	Non-experts
[16]	RBR	U Dys	Ward management of micturition	LUTS, Urinary tract infection Anatomical obstruction, Multiple causality and sensory impairment	Diagnosis and risk of fall	Multiple experts	Se 0.95, Sp 0.72, Likert scale Cronbach α 0.9	Urology ward nurses
[21]	FRB	U Dys	U dyn interpretation	U dyn variables	Detrusor and sphincter dysfunction	Not mentioned	Improve User Ac by 10%	Experts
[22]	ANN	U Dys	Uroflow interpretation	Value of slopes, frequency and value of maximums, ration of amplitude and total voiding time	Healthy or pathologic Uroflow	Patients data from U dyn	78 test cases ROC 0.7 Ac 79%	Experts
[23]	SVM	U Dys	Diagnosis	Age, examination, Uroflow, U dyn	Healthy or pathologic Uroflow	Patients data	Ac 84%, Se 93%, Sp 33%	Experts
[17]	FNM	U Dys	Diagnosis	46 defining Characteristics from NANDA-I	Diagnosis of U Dys	Multiple experts weighted the variables and literature review	kappa vs experts (0.92–0.42), Se 0.95, Sp 0.92	Experts and non-experts
[14]	FNM	CaP-BPD	Diagnosis of BPE and CaP	Clinical and pathological variables	CaP, BPE medical, BPE surgery	Patients data	10 folds CV AUC 0.86, se 100%, sp 98%	Non-experts
[24]	FRB	CaP-BPD	AP CP CaP BPE	LUTS, quality of life, fever, haematuria, haemospermia, painful ejaculation, fever, perineal pain, bone pain, pyuria, age, DRE	Diagnosis and treatment of prostatic disease	Multiple experts interviews, patients records and literature	Ac 0.76, Se 0.79, Sp 0.75, retrospective data (n = 105)	Residents, patients, medical students
[12]	FRB	CaP-BPD	AP CP CaP BPE	LUTS, quality of life, fever, haematuria, haemospermia, painful ejaculation, fever, perineal pain, bone pain, pyuria, age, DRE	Diagnosis and treatment	WEKA* to extract rules then experts to modify	200 test cases Ac 0.93, Se 0.97, Sp 0.99,	Residents, patients, medical students
[25]	RBR	CaP	Diagnosis before 1st biopsy	Age, race, FH, DRE, PSA, PSAD, PSAV, TRUS findings	Cancer and benign	Not mentioned	25 test cases Se 100% Sp 33% PPV 62%, NPV 100%	Experts
[13]	F-CBR	CaP	Radiotherapy dose for CaP	Gl, PSA, Distribution Volume Histogram	Radiotherapy dose	72 patients’ cases	Comparison to experts, Ac 85%	Experts
[26]	F-ONT	BPD	Diagnosis and treatment of BPE	LUTS, DRE	Watchful waiting, medical, surgery	Multiple experts weighted the variables	44 prospective cases, agreement kappa = 0.89	Experts and non-experts
[27]	RBR	S Dys	Diagnosis and treatment	Set of descriptors	Therapeutic dialogue	Not mentioned	10 Patients' evaluations	Couples
[28]	RBR	S Dys	Male S dys diagnosis	22 parameters from history and examination	ED diagnosis	GA rule extraction from 30 cases	Se (73–94%), Sp (78–96%) Ac (89%) vs Residents	Un specified
[29]	FRB	S Dys	Male S dys diagnosis and treatment	MH, non-coital erection, diabetes mellitus, coronary artery, neuropathies, sexual history, psychosocial history, depression, smoking, alcohol, examination, hormonal evaluation, cholesterol	Diagnosis and treatment of ED	Multiple experts’ interviews, Pearson analysis on variables from patients' data and literature	70 test cases vs experts and non-experts (Ac79%)	Non-experts
[30]	FNM	UTI	UTI treatment	Clinical data on UTI	Antibiotics course	Patients data and guidelines	Ac 86.8%, 38 random cases	Experts and non-experts
[31]	ANN	VUR	Decision support for intervention	Age, gender, number of UTIs prior to VUR diagnosis, UTI, of complete ureteral duplication noted on Ultrasound, the presence of bowel or bladder dysfunction	UTI or not	255 cases, 96 cases	AUC 0.76	Experts
[32]	ANN	Nlt	ESWL dose calculation	Age, stone size, stone burden, number of sittings	Number and power of shock	196 cases, 80 cases	coefficient of correlation 0.9	Experts

A total of 21 Expert Systems included supporting the decision making in Urological domains. Rule based reasoning was the most common model and urinary dysfunction was the commonest domain

*Prostate diseases* were represented in 6 systems while 3 of them modelled by [10, 12, 20] for diagnosing both benign and malignant prostatic disease, namely cancer prostate (CaP).

All systems in this domain were diagnosis support system with exception of [19] which also provided treatment for benign prostatic hyperplasia (BPH) and [11] calculated the required radiotherapy dose for treating CaP.

*Sexual dysfunctions* were modelled in 3 systems where [21] diagnosed male sexual dysfunction with an accuracy of 89%, while [22] added a therapeutic model for the same disease with an overall accuracy of 79%. Sexpert by [23] was the third system in this category developed in 1988 and in fact the oldest ES to be identified from our search in all urological domains. Interestingly this RB system was designed to interact directly with couples suffering from sexual dysfunction where the system responds to their query with a tailored therapeutic dialogue for treating their problem.

*Urinary tract infection (UTI)* was diagnosed and treated by one of the hybrid fuzzy systems FNM developed by [24] with an accuracy of 86.8%.

#### Diagnosis prediction

In this domain, ES quantifying the probability of a clinical diagnosis with a defined margin of error. They simulate a second expert opinion and it has been suggested that their use could eliminate unnecessary invasive investigation as the application of ANN by [26] could reduce up to 68% of repeated TRUS biopsies to diagnose CaP.

##### Domains

*Prostate cancer* was the main domain for this application with 19 systems out of 20. Most of them were designed to predict organ confinement before radical surgical excision of the prostate (Tables 3, 4). The target population were patients with clinically localised CaP and their accuracy reached high estimates as in [28], where the system was able to predict 98% of the low risk group for lymph node involvement using preoperative available date (PSA, clinical stage and Gleason score).

**Table 3:** Diagnosis prediction application of Expert Systems (ES) in Urology

Art	Mdl	Dom	Subdomain	Variables	Output	System training	Validation	Statistical outcome
[33]	ANN	CaP	Pre-biopsy diagnosis with TRUS variables	Age, PSA, number of biopsies, clinical diagnosis, PSAD, TRUS variables	Cancer or benign	N = 442 from single centre database	ROC AUC NPV, PPV ½ CV	NPV 97%, PPV 82% better than LR
[34]	ANN	CaP	Diagnosis PSA 2.5–4	Age, tPSA, creatinine phospho kinase, prostatic acid phosphatase	Cancer or benign	Multicentre data 522 (PSA 2.5–4)	ROC AUC CV 152 cases	AUC 0.74
[35]	ANN	CaP	Diagnosis PSA 4–10	Age, tPSA, %fPSA, TPV, DRE	Risk of cancer	656 data from Finnish trial	ROC, Sp, Se LOO	Se 79%, Sp 57%, Ac 62%, PPV 35, NPV 90
[36]	ANN	CaP	Diagnosis PSA 2–20	Age, tPSA, %fPSA, TPV, DRE	Risk of Cancer	1188 multi centre	ROC, Sp, Se, 1/10 CV	Sp 90%, Se 64%
[36]	ANN	CaP	Diagnosis in trial patients with PSA 4–10	Age, tPSA, %fPSA, TPV, DRE	Risk of Cancer	1188 multi centre	ROC, Sp, Se, 204 trial data PSA 4–10	Se 95%, Sp 23.3%, CI 17.4%–30.2%, P < 0.0002
[37]	ANN	CaP	Diagnosis	fPSA, TZD, PSAV, %f PSA, TZV, t PSA, and PSAD	Cancer or benign	PSA 2.5–4, 272 patients, multicentre data	ROC, AUC ¼ CV	AUC 0.88
[37]	ANN	CaP	Diagnosis	TZD, % f PSA, PSAD and TPV	Cancer or benign	PSA 4–10, 974 patients, multicentre data	ROC, AUC ¼ CV	AUC 0.91
[38]	ANN	CaP	Diagnosis after initial negative biopsy PSA 4–10	t PSA, %f PSA, TPV, TZV, PSAD, TZD	Cancer or benign	820 patients with PSA 4–10 European cancer detection studies	ROC AUC 1/3 CV	AUC 0.83
[39]	ANN	CaP	Diagnosis of BPE and CaP	Age, ethnicity, FH, IPSS, t PSA, %f PSA, DRE	Risk of cancer	Multicentre 354 patients, multicentre	ROC vs LR, 144 test set 40% CV	AUC ANN 0.8, LR 0.5
[40]	FRB	CaP	Early diagnosis	Age, t PSA, TPV	Risk of cancer	Experts aided in developing 77 fuzzy rules	Not published	None
[41]	ANN	CaP	Diagnosis PSA 2–10	Age, tPSA, %fPSA, TPV, TZV, PSAD, TZD = ANNA 1	Cancer and benign	228 data one centre	ROC, 30% CV	AUC 0.78
[41]	ANN	CaP	Diagnosis PSA 2–10	ANNA 1 + presumed circle area ratio and DRE	Cancer and benign	228 data one centre	ROC 30% CV	AUC 0.79, Sp 45%, Se 90%
[42]	ANN	CaP	Diagnosis	Age, tPSA, TPV, PSAD, DRE, and TRUS findings	Cancer and benign	3814 prostate cancer screening data	ROC AUC 1/3 CV, 2 centres prospective data	AUC: 0.74, 0.76, and 0.75 prospective 0.73, 0.74
[43]	ANN	CaP	Diagnosis	Age, DRE, PSA, PSAD, TZV, TZD = ANNA	Cancer and benign	TRUS, single centre 684 data	ROC AUC 1/4 CV	AUC 0.74
[43]	ANN	CaP	Diagnosis	ANNA + TRUS findings	Cancer and benign	TRUS, single centre 684 data	ROC AUC 1/4 CV	AUC 0.86
[44]	FNM	CaP	Diagnosis, PSA < 20	Age, PSA, %f PSA	Cancer and benign	1030 patients’ data, one centre	ROC, Sp, Se, 1/4 CV	AUC 0.8, Sp 52%, Se 90%
[45]	ANN	CaP	Prostate cancer early diagnosis PSA 4–10	Age, tPSA, %fPSA, TPV, DRE	Cancer or benign	606 multicentre group (PSA 4–10)	ROC AUC, 1/10 CV	AUC 0.83, AUC 0.74 in Finish group
[45]	ANN	CaP	Prostate cancer early diagnosis PSA 4–10	Age, tPSA, %fPSA, TPV, DRE	Cancer or benign	656 Finnish cancer survey group (PSA 4–10)	ROC AUC, 1/10 CV	AUC 0.77
[46]	ANN	CaP	Diagnosis	Age, DRE, t PSA and f PSA	Cancer and benign	1509 with PSA < 20, Single centre	ROC AUC, 1/5 CV	AUC 0.74
[46]	ANN	CaP	Diagnosis	Age, DRE, t PSA, f PSA, TPV and TRUS findings	Cancer and benign	1509 with PSA < 20, Single centre	ROC AUC, 1/5 CV	AUC 0.75
[47]	ANN	CaP	Diagnosis with -2 Pro PSA	Age, TPV, tPSA, %fPSA, p2 PSA, %p2 PSA (-2 proPSA)	Cancer and benign	PSA 1–30, 586 one centre	ROC, Sp, Se LOO 586	AUC 0.85, Sp 62%, Se 90%
[48]	ANN	CaP	Diagnosis pre-biopsy	Age, DRE, tPSA, PSAD, TZD, TRUS findings	Benign and malignant	600 patients with suspected CaP	ROC AUC, 477 random	AUC 0.77
[48]	SVM	CaP	Diagnosis pre-biopsy	Age, DRE, tPSA, PSAD, TZD, TRUS findings	Benign and malignant	600 patients with suspected CaP	ROC AUC, 477 random	AUC 0.85
[49]	ANN	CaP	Diagnosis PSA 2–20	Age, tPSA, %f PSA, DRE, TPV	Cancer and benign	Testing Prostataclass	ROC AUC, 165 patients one centre	AUC (PSA 2–10) 63–69%, (PSA 10–20) 57–88%
[50]	ANN	CaP	Diagnosis	Age, tPSA, %f PSA	Prognosis: cancer or not	121 Patients data from one centre	ROC AUC, 30% CV 29 patients	AUC 0.92
[51]	ANN	CaP	Diagnosis of clinically significant cancer	Age, DRE, PSA, PRV, TRUS, Biopsy cores	Disease clinical significance	3025 multicentre data	Accuracy estimation	Ac 57%
[52]	ANN	CaP	Diagnosis of cancer	Age, DRE, PSA, %fPSA, and TPV	Cancer and benign	204 PSA between 4 -10	ROC AUC	AUC 0.72
[53]	ANN	CaP	PHI index and TPV in diagnosis	Age, TPV, %fPSA, tPSA, PHI, %P2PSA	Cancer and benign	220 cases PSA < 10	ROC AUC	AUC 0.81
[53]	ANN	CaP	PHI index and TPV in diagnosis	Age, %fPSA, tPSA, PHI, %P2PSA	Cancer and benign	221 cases PSA < 10	ROC AUC	AUC 0.77
[54]	FRB	CaP	Diagnosis	Age, PSA, TPV	Cancer and benign	78 TRUS cases from Urology clinic	None	None
[55]	ANN	Fert	Sperm count	Age, duration of infertility, FSH, LH, TT and PRL, testicular volume	Presence of spermatozoa	303 patient’s data	ROC AUC then kappa stats of LR, test set 73 random	Se 68%, Sp 87.5%, PPV 73.9%, NPV 84%
[56]	ANN	Fert	Endocrinopathy with low sperm count	Testis volume, total sperm count,	Endocrinopathy	1035 Data from 2 centres	ROC AUC	AUC 0.95
[57]	ANN	Fert	Microdissection testicular sperm extraction	Age, FSH level, cryptorchidism and Klinefelter Syndrome	Sperm retrieval	1026 data, one centre	ROC AUC	Se 67% Sp 49.5% PPV, 63.9% NPV 52% Ac 60.8%
[58]	ANN	U Dys	Interpretation of U dyn and symptoms	Neurological and physical symptoms, flowmetry, cystometry, U dyn	Areflexia, hyper-reflexive, effort incontinence	400 U Dyn data	80 patients, 1/5 CV,	Accuracy 85%
[59]	ANN	U Dys	Interpretation of U dyn and symptoms	Neurological and physical symptoms, flowmetry, cystometry, U dynamics	Healthy or ill	300 patients with LUT disease	ROC, Ac, 1/5 CV	Accuracy 89%
[60]	ANN	U Dys	Bladder outlet obstruction	values of the average flow rate, Qmax, PVR and TPV	Obstructed, non-obstructed, and equivocal	N = 457 cases from single centre	Accuracy estimation 157 cases	Ac 60% (testing) 75% (training)
[61]	ANN	BPD	IPSS interpretation	IPSS subdomain scores	Obstructed, non-obstructed, and equivocal	N = 460 from single centre	Accuracy estimation 157 cases	Ac 73%

A total of 37 systems identified in this application of Expert Systems in Urology with evident prevalence of ANN as the model and CaP to be the dominant domain

**Table 4:** Disease stage prediction

Art	Mdl	Dom	Subdomain	Variables	Output	System training	Statistical outcome	Validation set
[62]	ANN	CaP	staging of localised disease	Age, race, DRE, tPSA, size of tumour on ultrasound, Gl, bilaterality of cancer and number of positive cores and perineural infiltration	Margin, seminal vesicle and lymph node positivity	1200, patients’ data from multicentre	AUC 0.77, 0.79, 0.8	20% CV
[63]	FSS	CaP	Localised disease staging	Age, PSA, PSAD, DRE, TRUS, Gl, CT, bone scan, chest x-ray, MRI	Localised or advanced	16 Cases	Se 92%, Sp 84%, Ac 82%	43 cases RRP
[64]	ANN	CaP	Lymph node staging in CaP post RPP	Age, Gl, clinical stage	Lymph node spread	736 data from one centre clinically localised CaP	Se 64%, Sp 81.5%, PPV 14%, NPV 98%	1840 and 316 cases from 2 centres
[65]	ANN	CaP	Prostate cancer staging post RRP	Age, tPSA, Gl, clinical stage	Lymph node spread or organ confinement	5744 data from one centre clinically localised CaP	AUC 77%, 88% for LN	25% CV random
[66]	ANN	CaP	Stage prediction post RRP	Age, histological variables from biopsy	CaP stage	97 cases with non-organ confined	Prediction accuracy ranged from 82 to 90%	
[66]	ANN	CaP	Stage prediction post RRP	Age, histological variables from biopsy, tPSA and TPV	CaP stage	77 cases with non-organ confined and extracapsular spread	Prediction accuracy ranged from 82 to 90%	
[67]	ANN	CaP	Prostate cancer staging post RRP PSA 2–10	tPSA, TNM, Gl (ANNA1)	localised disease	124 data from 2 centres Clinically localised CaP	AUC 0.82	20% (n = 36 patients)
[67]	ANN	CaP	Prostate cancer staging post RRP PSA 2–10	tPSA, TNM, Gl, maximum tumour length (ANNA2)	localised disease	124 data from 2 centres Clinically localised CaP	AUC 0.88	20% (n = 36 patients)
[67]	ANN	CaP	Prostate cancer staging post RRP PSA 2–10	tPSA, TNM, Gl, maximum tumour length, PSAD (ANNA3)	localised disease	124 data 2 centres Clinically localised CaP	Ac 83.3%, Se 85%, Sp 83%, PPV 73%, NPV 90% AUC 0.9	20% (n = 36 patients)
[67]	ANN	CaP	Prostate cancer staging post RRP PSA 2–10	tPSA, TNM, Gl, maximum tumour length PSAD, age (ANNA4)	localised disease	124 data 2 centres Clinically localised CaP	AUC 0.87	20% 36 patients
[68]	ANN	CaP	Prostate cancer staging post RRP	tPSA, TPV, TZV, PSAD, TZ, Gl	Pathological stage t2-4	201 cases from multinational European cancer data base (PSA 10 or less)	AUC 0.87	61 prospective set
[69]	ANN	CaP	diagnosis of skeletal metastasis	Age, tPSA	skeletal Mets	111 retrospective cases in one centre	AUC 0.88, Se 87.5%, Sp 83.3%	Bootstrap CV
[70]	ANN	CaP	Stage prediction post RRP	DRE, % of cancer, sum of tumour length, % cancer length and maximum cancer core length	advanced cancer (> pT3a)	300 randomly selected from retrospective data	AUC 0.71, Se 63%, Sp 81%, Ac78%	232 random selected set
[70]	SVM	CaP	Stage prediction post RRP	DRE, % of cancer, sum of tumour length, % cancer length and maximum cancer core length	advanced caner (> pT3a)	300 randomly selected from retrospective data	AUC 0.81, Se 67%, Sp 79%, Ac77%	232 random selected set
[71]	ANN	CaP	Define precise stage	PSA, clinical stage, pathological stage, Gl (other added for different set: erection, IPSS, TRUS size, MRI stage	margin, seminal vesicle and lymph node positivity	From 7500 patients’ data from BAUS database and remodelled with external data of 85 patients	AUC 0.38–0.67, concordance index for variables	10 folds CV
[71]	BN	CaP	Define precise stage	PSA, clinical stage, pathological stage, Gl (other added for different set: erection, IPSS, TRUS size, MRI stage	margin, seminal vesicle and lymph node positivity	From 7500 patients’ data from BAUS database and remodelled with external data of 85 patients	AUC 0.01–0.67 concordance index for variables	10 folds CV
[71]	kNN	CaP	Define precise stage	PSA, clinical stage, pathological stage, Gl (other added for different set: erection, IPSS, TRUS size, MRI stage	margin, seminal vesicle and lymph node positivity	From 7500 patients’ data from BAUS database and remodelled with external data of 85 patients	AUC 0.33–0.6 concordance index for variables	10 folds CV
[71]	RBF	CaP	Define precise stage	PSA, clinical stage, pathological stage, Gl (other added for different set: erection, IPSS, TRUS size, MRI stage	margin, seminal vesicle and lymph node positivity	From 7500 patients’ data from BAUS database and remodelled with external data of 85 patients	AUC 0.45–0.5 concordance index for variables	10 folds CV
[71]	SVM	CaP	Define precise stage	PSA, clinical stage, pathological stage, Gl (other added for different set: erection, IPSS, TRUS size, MRI stage	margin, seminal vesicle and lymph node positivity	From 7500 patients’ data from BAUS database and remodelled with external data of 85 patients	AUC 0.5 concordance index for variables	10 folds CV
[72]	ANN	CaP	Staging post RRP	Age, tPSA, n Positive cores, involvement per core, % of positive core	Organ confinement and metastasis	870 multicentre data	Ac 60%	120 cases, Accuracy estimation
[73]	FNM	CaP	Cancer staging of organ confinement	Age, PSA, Primary Gleason Pattern, secondary Gleason pattern, clinical stage	Organ confinement and metastasis	399 cases from research network database	AUC 0.8, FNM outperformed ANN, FCM, LR	ROC AUC vs other models
[74]	ANN	Nsc	staging	vascular, lymphatic, tunical invasion, percentage of embryonal carcinoma, yolk sac carcinoma, teratoma and seminoma	Stage one or two	93 cancer specimen, single centre	Prediction accuracy 79.6 to 87.1%,	10 folds CV

This table demonstrated Expert Systems predicting urological diagnosis from variable clinical and radiological date. Artificial neural networks (ANN) diagnosing localised prostate cancer (CaP) before surgery were the most common systems in this application

Chiu et al. [29] modelled a system with clinical variables for patients undergoing nuclear bone scintigraphy for predicting skeletal metastasis. The system was able to predict metastatic disease in the test group with Se 87.5%, Sp 83.3%.

*None seminoma testicular cancer* was the other domain in this application with the system [27] able to predict the cancer disease stage (Table 4) with accuracy reaching 87%.

#### Treatment outcome prediction

In this application, ES combined disease and patient related factors to estimate the success of a specific treatment or intervention. As in [30, 38, 64, 69] where the system predicted the outcome of extra corporeal shock wave (ESWL) for treating kidney stones and [74, 75] providing an estimation of cancer recurrence after radical surgical treatment of prostate cancer.

##### Domains

*Prostate cancer* was also common domain in this application (n = 23). Potter [74, 75] described 4 models developed by data acquired from patients with clinically localised CaP and had radical prostatectomy with curative intent. The variables included clinical and histological findings of the surgical specimen and they were able to predict up to 81% who did not have evidence biochemical failure (rising PSA) in their follow up. Hamid et al. [76] and Gomha [77] models were not restricted to the clinically localised CaP cohort and their study population included patients at different disease stages and on any treatment pathway. Their models included 2 experimental histological markers (tumour suppressor gene p53 and the proto-oncogene bcl-2) in their input variables and the estimated predictive accuracy of the patient response to treatment were reaching 68% and 80% (*p* < 0.00001) respectively.

*Nephrolithiasis treatment* was expressed by 6 other systems applying the treatment outcome prediction concept. Cummings et al. targeted this group in his ANN [78] where he trained his network with patients’ data treated at the emergency service of 3 centres with ureteric stones, to identify patients failing conservative management and requiring further intervention. When tested on a different set of 55 cases, the system correctly predicted 100% of the patients who passed the stone spontaneously with an overall accuracy of 76%.

Extra corporeal shockwave lithotripsy (ESWL) is one of the favourable interventions in the nephrolithiasis treatment domain. The stone here receives strong external shock waves, which can subsequently reduce it into small fragment and eliminate the need for direct instrumentation of the renal tract. Their reported success rate can only provide a generalised prediction of outcome to the individual case and ANN was capable of providing an alternative multivariate analytical tool in the 4 models developed by [30, 38, 64, 69]. They estimated high accuracy of their models (Table 5), as in [64], the system predicted 97% of the patients who were confirmed to be stone free following ESWL for treating ureteric stone.

**Table 5:** Treatment outcome prediction

Art	Mdl	Dom	Subdomain	Variables	Output	System training	Validation methods	Statistical outcome
[79]	ANN	CaP	Outcome of RRP	Age, stage, bone scan, grade, PSA, treatment, bcl-2, p54	No response, response then relapse, response and no relapse	cohort of CaP single centre 21 patients	ROC, Sp, Se 20 patients randomly selected	Ac 85% (60% without markers), K, 0.65; Cl, P < 0.00001
[80]	ANN	CaP	BCF post RRP	Age Pathologic findings and GENN1	Disease progression	Gl 5–7, T1B-2C, Single centre 136	ROC, Sp, Se Test set of 35 (20%)	AUC 0.71, Ac 74%, Se 82%, Sp 61%,
[80]	ANN	CaP	BCF post RRP	DNA polyploidy and quantitative nuclear grade	Disease progression	Gl 5–7, T1B-2C, Single centre 136	ROC, Sp, Se	AUC 0.74, Ac 80%, Se 75%, Sp 85%
[80]	ANN	CaP	BCF post RRP	Pathologic findings, age, DNA polyploidy and quantitative nuclear grade	Disease progression	Gl 5–7, T1B-2C, Single centre 136	Test set of 35 (20%)	AUC 0.73, Ac 78%, Se 84%, Sp 72%
[81]	ANN	CaP	BCF post RRP	Age, PSA, Gl and stage	BCF post RRP all	140 cases post RRP, one centre	ROC, Sp, Se 35 (20%) for validity	AUC 0.81, Se 74%, Sp 78%, PPV 71%, NPV 81%,
[82]	Fkn	CaP	Outcome of RRP	TM, Gl, PSA, P53, bcl-2, treatment method	No response No progression after treatment, Relapse	41 men with CaP	LOO and compare predictive accuracy of ANN, Fkn	Predictive accuracy ranged from 61–88%
[68]	ANN	CaP	Outcome of RRP	tPSA, TZV, PSAd, Gl	Local or advanced disease	200 cases from multinational European cancer data base	AUC ROC 60 prospective set	AUC 0.91, Se 95%, Sp 64%,
[83]	ANN	CaP	Outcome of RRP, margin positive	tPSA, clinical stage, Gl (ANNA1)	Positive surgical margins	218 post RRP and pelvic lymph adenectomy in one centre	ROC AUC 48 cases 1/4 CV	AUC 0.7
[83]	ANN	CaP	Outcome of RRP, margin positive	tPSA, clinical stage, Gl, pMRI findings (ANNA2)	Positive surgical margins	218 post RRRP and pelvic lymph adenectomy in one centre	ROC AUC 48 cases 1/4 CV	AUC 0.87
[83]	ANN	CaP	Outcome of RRP, margin positive	tPSA, clinical stage, Gl, pMRI findings, % of cancer in biopsy, PSAd ANNA3	Positive surgical margins	218 post RRP and pelvic lymph adenopathy in one centre	ROC AUC 48 cases 1/4 CV	AUC 0.87
[83]	ANN	CaP	Outcome of RRP, margin positive	tPSA, clinical stage, Gl, % of cancer in biopsy ANNA4	Positive surgical margins	218 post RRP and pelvic lymph adenopathy in one centre	ROC AUC 48 cases 1/4 CV	AUC 0.71
[84]	ANN	CaP	Outcome of RRP, margin and LN	tPSA, clinical TNM Gl ANNA1	Positive surgical margins, LN involvement	41 post RRP and pelvic lymph adenopathy in one centre	ROC AUC 160 cases randomly selected	AUC 0.86 for positive margin, 0.88 for LN + ve
[84]	ANN	CaP	Outcome of RRP, margin and LN	tPSA, clinical TNM Gl, pMRI findings ANNA2	Positive surgical margins, LN involvement	41 post RRP and pelvic lymph adenopathy in one centre	ROC AUC 160 cases randomly selected	AUC 0.9 for positive margin, 0.89 for LN + ve
[84]	ANN	CaP	Outcome of RRP, margin and LN	tPSA, clinical stage, Gl, pMRI findings, age ANNA3	Positive surgical margins, LN involvement	41 post RRP and pelvic lymph adenopathy in one centre	ROC AUC 160 cases randomly selected	AUC 0.9 for positive margin, 0.9 for LN + ve
[85]	FRB	CaP	Outcome of RRP	Clinical stage, Gl, tPSA	Cancer stage (confined, capsule, vesicle and LN)	116 rules developed from nomograms	ROC Se, Sp 190 patients post RRP in one centre	AUC 0.76 (95% CI 0.7–0.8), Se 85%, Sp 61%)
[86]	ANN	CaP	Outcome of RRP, margin positive	TNM stage, age, Gl, tPSA	Capsule penetration	650 retrospective data for RRP at one centre	PPV, NPV 98 cases for testing and 1/2 CV	PPV 100%, NPV 95%
[86]	ANN	CaP	Outcome of RRP, margin positive	TNM stage, age, Gl, tPSA MLP	Capsule penetration	650 retrospective data for RRP at one centre	PPV, NPV 98 cases for testing and 1/2 CV	PPV 97%, NPV 95%
[86]	ANN	CaP	Outcome of RRP, margin positive	TNM stage, age, Gl, tPSA, Partial RNN (recurrent neural network)	Capsule penetration	650 retrospective data for RRP at one centre	PPV, NPV 98 cases for testing and 1/2 CV	PPV 97%, NPV 95%
[86]	ANN	CaP	Outcome of RRP, margin positive	TNM stage, age, Gl, tPSA, RBF-MLP	Capsule penetration	650 retrospective data for RRP at one centre	PPV, NPV 98 cases for testing and 1/2 CV	PPV 97%, NPV 94%
[87]	FRB	CaP	Outcome of RPP	Clinical stage, Gl, tPSA	Capsule penetration	Genetic algorithm on 331 patients post RRP in one centre	48 patients post RRP in one centre ROC	AUC 0.82 (95% CI 0.5–0.8)
[88]	ANN	CaP	Outcome of LAP RRP, BCF	Clinical and pathologic parameters, tPSA, margin status, TNM and Gl	BCF	1575 patients at one centre post lap RRPP	ROC AUC LOO	AUC 0.75, Se 90%, Sp 35
[32]	FNM	CaP	Outcome post RRP	Age, FH, DRE, tPSA, Gl, MR findings	tPSA at 6 months	19 one centre post RRP	Correlation coefficient = 0.99	3 Cases
[89]	ANN	CaP	Outcome post RRP	Age, tPSA, staging, perineural infiltration, Gl, months of FU	BCF	1400 multicentre data	Se 85% Sp74%, PPV 77%	400 data
[90]	ANN	CaP	Outcome post RRP, organ confined	Gleason score, preoperative PSA and clinical stage,	Organ confined	468 cases for training	NPV 83%	47 cases 30% CV
[91]	ANN	CaP	Outcome of RRPP	PSA, BMI, DRE, TRUS, Gl score or grade	Capsule penetration	225 patients’ data post RRP from 3 centres	74 patients randomly selected ROC	AUC 0.79 LR 0.74 (P = 0.016) Partin AUC 0.7
[78]	ANN	Nlt	Stone regrowth after ESWL	Anatomy, position, stone analysis, urine analysis, previous stone, medical treatment	Stone recurrence	single centre data base, 65 cases	ROC, Sp, Se33 cases	Se 91%, Sp 92%, AUC 0.96
[92]	ANN	Nlt	Stone clearance with conservative treatment	Age, gender, duration, creatinine, nausea, vomiting, fever	Clearance or intervention	multi centre, Ureteric stone 125 cases	55 cases ROC, Sp, Se	AC 76% Predict 100% of stones passed
[75]	ANN	Nlt	lower pole stone ESWL	Gender, BMI, radiology, stone size and composition, urine analysis, 24 h urine, serum ca and creatinine	Clearance or intervention	321 patients with lower pole stone	211 random set ROC, Sp, Se, vs LR	AUC 0.97 Se 95%, Sp 92%,
[76]	ANN	Nlt	Stone clearance with ESWL	Age, gender, body habitus, serum electrolytes, 24 h urine, radiological findings	Stone free	60 patients, one centre	Correlation co-efficient 22 cases	0.75
[77]	ANN	Nlt	Stone clearance with ESWL	Age, gender, anatomy, location, side, number, length, width, new or recurrent, stent	Stone clearance	Ureteric stone ESWL, One centre 688 cases	296 cases ROC, Sp, Se	Ac 78%, Se78%, Sp 75%, PPV 97%
[93]	ANN	Nlt	Outcome of conservative stone disease treatment	Age, gender, BMI, fever, previous treatments and stones, duration of the symptoms, dimension and position of the stone	Spontaneous expulsion or intervention	402 patients from one centre	50 patient, 1/4 cross validation ROC Se, Sp	Se 95%, Sp 63%
[93]	SVM	Nlt	Outcome of conservative stone disease treatment	Age, gender, BMI, fever, previous treatments and stones, duration of the symptoms, dimension and position of the stone	Spontaneous expulsion or intervention	402 patients from one centre	50 patient, 1/4 cross validation ROC Se, Sp	Se 85%, Sp 87%
[94]	ANN	Nlt	ESWL outcome prediction	The patients’ characteristics, stone location, burden, shape dimension, pre-ESWL procedure and cost of admission	unexpected post-ESWL visits	1026 patients received ESWL at one centre`	AUC 0.66	506 patients
[95]	ANN	PUJ	Outcome of PUJ repair	Demographic, clinical and radiological findings	Sonographic outcome of pyeloplasty	Single centre unilateral paediatric pyeloplasty n = 100	16 cases (16%) ROC, Sp, Se	Ac 100%, Se 100%, Sp 100%
[96]	ANN	PUJ	Outcome of PUJ conservative treatment	Age, gender, renal pelvis diameter, laterality, separated renal function on DMSA, urine culture and infections	Observation or surgery	37 infants with PUJ obstruction	Prediction accuracy16 patients for validation	75% prediction accuracy
[97]	ANN	Neph	Post lap partial nephrectomy hospital stay	Age, co-morbidities, tumour size and extension	Hospital stay less than 2 days	334 one centre	5 institutes 77, 19 prospective ROC	AUC 0.6, 0.5
[97]	ANN	Neph	Post lap nephrectomy hospital stay	Age, co-morbidities, tumour size and extension	Hospital stay less than 2 days	392 One centre	5 institutes 127, 29 prospective ROC	AUC 0.7, 0.7
[98]Z	ANN	Bca	Pathological stage after surgery	Age, gender, tumour (size, number, grade, invasion, lymph vascular invasion, stage), lymph nodes	Prognosis and advanced stage	183 patients, one centre post cystectomy	ROC and compare with LR 1/3 cross validation	MANN AUC 0.86, Se 88%, Sp 77%, PPV 93%, NPV 63%, Ac 85%
[98]	ANN	Bca	Pathological stage after surgery	Age, gender, tumour (size, number, grade, invasion, lymph vascular invasion, stage), lymph nodes	Prognosis and advanced stage	183 patients, one centre post cystectomy	ROC and compare with LR 1/3 cross validation	SANN AUC 0.85, Se 84%, Sp 71%, PPV 91%, NPV 67%, Ac 83%
[99]	ANN	VUR	outcome of endo repair of VU reflux	Age, gender, implant type, implant volume, number of treatments, side, endo findings, type of cystography	Ultrasound finding	Single centre data base, paediatric VU reflux 174 data	87 cases for validation ROC, Sp, Se	Se 71.4%, Sp 81.6%, PPV 58.8%, NPV 88.6% and success rate 78.9%,

Is one of the common applications of urological expert system. They predicted treatment outcome of radical nephrectomy, radical cystectomy, radical prostatectomy, vesico ureteric reflux endoscopic repair, pelvi-ureteric junction obstruction conservative management, nephrolithiasis conservative management and extracorporeal shockwave treatment. The commonest domain was predicting negative surgical margins post radical prostatectomy

*Paediatric pelvi-ureteric junction obstruction* is primarily treated conservatively unless there is any evidence of renal function compromise, recurring infection or worsening radiological findings. For the failing group, pyeloplasty is the second line of treatment and [81] developed an ANN to estimate the success rate of this procedure for each individual case by predicting the post-operative degree of hydronephrosis with a reported 100% accuracy in the small tested sample.

*Vesico ureteric reflux or reflux uropathy* is another paediatric disease, characterised by back flow of urine from the bladder into the ureter through incompetent Vesico ureteric functional valve. Treatment is primarily conservative as it can be a self-limiting disease or surgery to reimplantation the ureters or endoscopic injection of bulking agent at the ureteric orifices [80]. The study authors trained a neural network using 261 cases whom have received endoscopic injection and the system predicted 94% of the patients who did not benefit from the treatment [80].

*Laparoscopic partial and radical nephrectomy* were the domain of the [82], which was developed by multi institutional case data (age, co-morbidities, tumour size, and extension) of patients having laparoscopic partial or radical nephrectomy. The system was able to predict the length of their postoperative hospital stay with an accuracy of 72%.

*Bladder cancer* can be treated with complete bladder excision and [79] developed systems to predict the cure rate with an accuracy of 83%.

#### Recurrence and survival prediction

The ES in this domain aimed to provide individualised risk analysis tools estimating the disease specific mortality and recognising the group whom may benefit from more aggressive or adjuvant treatment.

##### Domains

*Bladder cancer* survival and recurrence prediction following radical cystectomy (RC) with curative intention was the commonest domain in this application (24 out of 26 total systems). The lymph nodal involvement is highly predictive of the recurrence and these patients are considered for adjuvant or neoadjuvant systemic chemotherapy. The node free cohort will include high-risk patients who were not identified by the conventional linear stratification system. Catto et al. developed a FNM system to identify this high risk group in the nodal free cohort by predicting the disease recurrence rate (Se 81%, Sp 85%) and their survival with a median error of 8.15 months [92]. The high-risk group identified by this model can benefit from systemic treatment post cystectomy to improve their disease related morbidity and mortality [95, 96]. The 5 years survival post cystectomy was the output of 2 other ANN with a high prediction efficacy of 77% and 90% respectively (Table 6) [97, 99].

**Table 6:** Recurrence and progression prediction

Art	Mdl	Dom	Subdomain	Variables	Output	Knowledge acquisition	Validation	Statistical outcome
[83, 100]	ANN	Bca	Recurrence	Age, gender, smoking, tumour stage and grade, CIS, number, cytology, other mucosal biopsy	Recurrence or no	N = 432 patients’ data, multicentre	Radom set of 200 ROC AUC	Se 76%, Sp 55%, Ac 72%
[101]	ANN	Bca	Tumour progression recurrence	Tumour stage and grade, size, number, gender, eGFR	Stage progression	105 Ta/T1 TCC multicentre	Compare to 4 clinicians McNemar test	80% accuracy
[101]	ANN	Bca	12 months cancer specific survival	Tumour stage and grade, size, number, gender, eGFR, smoking, cis, dysplasia tumour site, architecture, c-erbB2 (oncogene), p53 (tumour suppressor gene)	6 months recurrence 12 months survival	56 Ta/T1 (6 months recurrence), 40 T2-T4 (12 months survival)	Compare to 4 clinicians McNemar test	Accuracy to predict recurrence (75%) and to predict survival (82%)
[102]	ANN	Bca	Progression of non-invasive TCC	Age, gender, tumour (grade, stage, number and architecture) and mean nuclear volume	Tumour progression and recurrence	68 patients’ specimen from one centre	22 Random test set ROC, Sp, Se	Recurrence: Se 33%, Sp 40%, PPV 40%, NPV 33% Progression: Se 100%, Sp 67%, PPV 40%, NPV 100%
[103]	FNM	Bca	Recurrence classifier	Age, gender, grade, smoking, previous cancer, p53, hMLH1, hMSH2	Recurrence or not	109 patients from one centre with TCC	10% cross validation ROC, LR	AUC 0.98, Se 90%, Sp 80%, PPV 92%, NPV 74%, Ac 88%
[103]	FNM	Bca	Survival predictor	Age, gender, grade, smoking, previous cancer, p53, hMLH1, hMSH2	Survival in months	109 patients from one centre with TCC	10% cross validation Root mean square	RMS = 4.8
[103]	ANN	Bca	Recurrence classifier	Age, gender, grade, smoking, previous cancer, p53, hMLH1, hMSH2	Recurrence or not	109 patients from one centre with TCC	ROC, LR 10% cross validation	AUC 0.91, Se 94%, Sp 96%, PPV 99%, NPV 84%, Ac 95%
[103]	ANN	Bca	Survival predictor	Age, gender, grade, smoking, previous cancer, p53, hMLH1, hMSH2	Survival in months	109 patients from one centre with bladder	10% cross validation RMS	RMS = 11.7
[104]	ANN	Bca	Survival predictor	Age, stage, Grade, smoking, previous cancer	Risk of relapse	109 patients with primary TCC	Difference in RMS 1/4 CV ROC AUC	Se 90%, Sp 89%, PPV 98, NPV, 64%, Ac 90%, RMS 8.8
[104]	ANN	Bca	Recurrence predictor	Stage, Grade, age, smoking, previous cancer, p53, hMLH1, hMLH2	Time to relapse	109 patients with primary TCC	Difference in RMS 1/4 CV ROC AUC	Se 94, Sp 96%, NPV 99%,PPV 84%, Ac 95%, RMS 7.6
[104]	FNM	Bca	Survival predictor	Stage, Grade, age, smoking, previous cancer	Risk of relapse	109 patients with primary TCC	Difference in RMS 1/4 CV ROC AUC	Se 92%, Sp 90%, PPV 98% NPV 72%, Ac 92%, RMS 8.5
[104]	FNM	Bca	Recurrence predictor	Stage, Grade, age, smoking, previous cancer, p53, hMLH1, hMLH2	Time to relapse	109 patients with primary TCC	Difference in RMS 1/4 CV ROC AUC	Se 90% Sp 80%, NPV 92%,PPV 74%, Ac 88%, RMS 7.3
[105]	FNM	Bca	Recurrence (classifier)	Age, gender, grade, smoking, previous cancer, p53, methylation index (% of loci on chromosomes), RARB methylation (gene locus)	Recurrence or not	117 patients with 1ry TCC or UCC from one centre	10% cross validation ROC, LR	AUC 0.98, Se 88–100%, Sp 94–100%, Ac 100%
[105]	FNM	Bca	Survival predictor	Age, gender, grade, smoking, previous cancer, p53, methylation index (% of loci on chromosomes), RARB methylation	Survival in months	117 patients with 1ry TCC or UCC from one centre	10% CV Kaplan Maier for survival	Average error = 5 months
[105]	ANN	Bca	Recurrence (classifier)	Age, gender, grade, smoking, previous cancer, p53, methylation index (% of loci on chromosomes), RARB methylation	Recurrence or not	117 patients with 1ry TCC or UCC from one centre	10% cross validation ROC, LR	Ac 89–90%, Se 81–87%, Sp 95–100%
[105]	ANN	Bca	Survival predictor	Age, gender, grade, smoking, previous cancer, p53, methylation index (% of loci on chromosomes), RARB methylation	Survival in months	117 patients with 1ry TCC or UCC from one centre	10% CV Kaplan Maier for survival	Average error = 9 months
[106]	ANN	Bca	Recurrence	Age, sex, previous recurrence, response to adjuvant therapy, number of lesions, adjuvant therapy	Recurrence or no	403 patients	1/3 CV 123 patients ROC AUC	AUC 0.87,Se 79%, Sp 98%
[107]	ANN	Bca	5 Years survival cystectomy	Age, gender, tumour stage, grade, ln, vascular in, perineural in, prostatic invasion, CaP	Survival at 5 years	369 patients	ROC, Cox proportional hazard 1/3 CV	Se 63%, Sp 86%, PPV 76%, NPV 77%
[108]	FNM	Bca	Recurrence classifier	Gender, pathological stage, grade, CIS, lymph vascular invasion	Recurrence or not	609 patients from multiple centres	ROC, LR 10% CV	Se 93%, Sp 68%
[108]	FNM	Bca	Survival predictor	Gender, pathological stage, grade, CIS, lymph vascular invasion	Survival in months	172 multicentre data	ROC, LR 10% CV	Kaplan–Meier survival plots, median error of 8.15 months
[109]	ANN	Bca	Survival post cystectomy	Age, gender, bilhariziasis, histology, grade, lymph nodes, lymph vascular, type of diversion	Patient survival	871 patients’ data post cystectomy	30% CV ROC vs LR	AUC 0.86, Se 79%, Sp 81%
[110]	ANN	Bca	bladder cancer 5 years survival	Age, gender, histology grade, tumour stage, positive LN, removed LN	5 years survival	cystectomy data base, single centre 106 patients	Prediction error percent 11 and 29 patients	prediction error rate, > 90% efficiency
[111]	ANN	Bca	Recurrence and survival	Age, gender, tumour stage, grade, CIS, ln, lymph vascular invasion	5 years recurrence and cancer specific death	cystectomy data base, multicentre 2111	ROC, Kaplan Maier for survival, Cox Proportional Hazard	Se 59%, Sp 77%, PPV 67%, NPV 70% (30% cross validation)
[112]	ANN	Bca	Survival post cystectomy	Age, gender, albumin, surgical approach, tumour stage, follow up period, type of diversion	5 years survival	117 patients with post cystectomy from one centre	10 Folds CV ROC, Se, Sp Ac	Ac 72–80% RELM and ELM had best performance
[113]	ANN	Bca	Recurrence of G3 pTa after TURBT	Age, sex, previous histopathological data, previous recurrence rate response to previous BCG adjuvant therapy, number of lesions, size of lesions presence of inflammatory reaction and adjuvant therapy	Recurrence or No	143 patients with G3 pTa at one centre	AUC, Se, Se 1/3 cv 43 cases	AUC 0.81, Se 82%, Sp 96%
[114]	ANN	RCC	RCC survival 36 months	Age, gender, BMI, performance status, histopathology, time interval between primary tumour and detection of Mets, type of systemic therapy, number and sites of Met	Recurrence within 36 months	175 single centre	30% CV ROC sensitivity analysis	AUC 0.95 (95% CI 0.87–0.98)
[115]	ANN	Nsc	Disease recurrence in five years	(32 variables) age, tumour type, grade, invasion, Mets, ln, treatment, FBC, kidney function	Recurrence within five years	202 multicentre cases	1/4 CV ROC, Sensitivity analysis	AUC 0.87
[116]	FNM	CaP	Prognosis and survival	Age, BMI, PSA, DRE, Gl, clinical stage and treatment methods	Disease specific survival	100 cases single centre	Sensitivity analysis mean square error,	MSE = 0.068907 (1/10 CV)
[117]	ANN	Nlt	Recurrence of Upper tract stones	Age, sex, history of previous calculi, radiologic type, location and composition of previous calculi, 24-h urine assay urine culture, treatment	Recurrence of Upper tract stones	168 cases, single centre	PPV estimation 68 cases	PPV 100%

The majority of the Expert Systems in this application were artificial neural network predicting recurrence and survival following bladder cancer treatment. Other systems were applied in non-seminomatous testicular cancer, prostate cancer, renal cell carcinoma and recurrence of upper renal tract stones

*Renal cell cancer is* primarily treated with partial or radical nephrectomy for clinically localised disease with systemic therapy for the metastatic disease. There is still a degree of uncertainty in stratifying individual disease risk in order to predict the indication and outcome of systemic therapy in the group with distant metastasis. Vukicevic et al. [98] attempted to clarify this uncertainty by training a neural network with patients’ data who had nephrectomy (partial or radical) and received systemic therapy. The mature model predicted the patients who survived the disease at 3 years with an overall accuracy of 95% (CI 0.878–0.987).

*None seminoma testicular cancer* 5 years recurrence was the domain of [118] ANN. The system was trained with multicentre data and in its testing phase and predicted 100% of the patients who did not suffer from disease recurrence at 5 years with an overall predictive accuracy of 94% (AUC = 87%).

#### Predicting research variables

In academia, testing a hypothesis for ‘factors-outcome effect’ is a popular quest and the standard statistical regression analysis tools may not be effective for data contaminated by irrelevant variables [119]. AI can provide an alternative methodology in the analysis to identify variables with high correlation to the outcome by applying machine learning as in ANN. The area under the curve (AUC) is estimated for the system predictive accuracy applying all researched variables. Those research variables can be given random values or randomised then the AUC is re estimated for comparison with the original [120]. Only variables that decreases the AUC are considered significant and the wider the discrepancy of the AUC the more significant they are (Table 7).

**Table 7:** Research variable prediction

Art	Mdl	DOM	Subdomain	Variables	Output	System training	Validation	Statistics	Research outcome
[121]	ANN	BPE/CaP	Analysis of variables of quality of life questionnaire	Questionnaire suggested by medical and allied professional	High- or low-quality group	Single centre recruitment with BPE or CaP, 63 cases	ROC, Linear quadratic and logistic regression	Ac 90%, Se 94%, Sp 85%, PPV 89%, NPV 92%	Identify relevant variables
[78]	ANN	Nlt	Stone recurrence after ESWL	Anatomy, position, stone analysis, urine analysis, previous stone, medical treatment	Stone recurrence	65 patients post ESWL from single centre	33 test set ROC AUC vs LR	AUC 0.96, Se 91%, S 91%	Stone recurrence, fragments not risk factor
[122]	ANN	CaP	Biochemical failure post RRP	TNM, tPSA, Gleason, pathology stage	BCF at 3 years Yes or no	564 patients’ data post RRRP with Gl 7, single centre	ROC, Kaplan Meier and Cox Proportional Hazards Model	AUC 75%, NPV 84	Gleason 7 is inversely correlated to disease free survival and direct to BCF
[122]	ANN	CaP	Biochemical failure post RRP	TNM, tPSA, Gleason, pathology stage	BCF post RRRP	564 patients’ data post RRRP with clinically localised CaP Gl7, single centre	ROC, Kaplan Meier for survival and Cox Proportional Hazards	AUC 81%, NPV 93%	
[75]	ANN	Nlt	lower pole stone ESWL	Gender, BMI, radiology, stone size, composition, urine analysis, 24 h urine, serum ca and creatinine	Clearance or intervention	321 patients with lower pole stone	211 random set ROC, Sp, Se, vs LR	AUC 0.97, Se 95%, Sp 92%	BMI, normal urinary transport and infundibular width of 5 mm or more and the infundibular ureteropelvic angle is 45° or more are correlated with stone clearance
[103]	FNM	Bca	Recurrence classifier	Age, gender, grade, smoking, previous cancer, p53, hMLH1, hMSH2	Recurrence or not	109 patients from one centre with bladder TCC	tenfold CV ROC, LR	AUC 0.98, Se 90%, Sp 80%, PPV 92%, NPV 74%, Ac 88%	p value calculated to compare all models, the effect of combining HK p53 with other variables
[103]	FNM	Bca	Survival predictor	Age, gender, grade, smoking, previous cancer, p53, hMLH1, hMSH2	Survival in months	109 patients from one centre with bladder TCC	tenfold CV Root mean square	RMS = 4.8	
[103]	ANN	Bca	Recurrence classifier	Age, gender, grade, smoking, previous cancer, p53, hMLH1, hMSH2	Recurrence or not	109 patients from one centre with bladder TCC	ROC, LR 10% cross validation	AUC 0.91, Se 94%, Sp 96%, PPV 99%, NPV 84%, Ac 95%	
[103]	ANN	Bca	Survival predictor	Age, gender, grade, smoking, previous cancer, p53, hMLH1, hMSH2	Survival in months	109 patients from one centre with bladder TCC	10% cross validation RMS	RMS = 11.7	
[123]	ANN	Bca	diagnosis	Urine levels of nuclear matrix protein-22, monocyte chemoattractant protein-1 and urinary intercellular adhesion molecule-2	Cancer and benign	253 Data from one cystoscopy clinic	ROC, Sp, Se	Se 100%, Sp 75.7%, PPV 32.9%, NPV 100%,	The three factors improve diagnosis
[124]	ANN	BPE	Significant LUT symptoms in BPE	Age, PSA, Qmax, TZV, TPV, Oss, ISS, PVR	Progression or no	397 patient with mild LUTS from 4 centres	1/3 CV ROC, Sp, Se, Then sensitivity analysis	Ac 79%, Se 82%, Sp 77%, PPV 78%, NPV 81%	PSA, Oss, TZV are correlated to disease progression
[125]	ANN	Hgon	Diagnosis of hypogonadism,	Age, ED, depression score, sexual health score, testosterone level	Risk of hypogonadism	148 one centre	70 test cases		Depression most significant, p < 0.0019
[126]	ANN	BPE/CaP	Diagnosis of BPE and CaP	Age, tPSA, %f PSA, TPV, MIC-1, Hk11, MIF	Cancer and benign	Single centre 371 patients	LOO	AUC 0.91, Se 90%, Sp 80%	Positive if all makers added together
[127]	ANN	Bca	Survival and recurrence predictor	22 different genes variables	Risk and time to relapse	67 bladder neoplasms and 8 normal bladder specimens	Difference RMS 10 folds CV ROC AUC	RMS 5.2 Ac 100%	500 genes where reduced to 22 genes for creating the network, thus significant
[127]	FNM	Bca	Survival and recurrence predictor	66 rules from 11 gene variables	Risk and time to relapse	67 bladder neoplasms and 8 normal bladder specimens	Difference RMS 10 folds CV ROC AUC	RMS 2.2 Ac 100%	500 genes where reduced to 22 genes for creating the network, thus significant
[105]	FNM	Bca	Recurrence (classifier)	Age, gender, grade, smoking, previous cancer, p53, methylation index (% of loci on chromosomes), RARB methylation	Recurrence or not	117 patients with 1ry TCC or UCC from one centre	10% cross validation ROC, LR	AUC 0.98, Se 88–100%, Sp 94–100%, Ac 100%	p value calculated to compare all models, the effect of combining HK p53 with other variables
[105]	FNM	Bca	Survival predictor	Age, gender, grade, smoking, previous cancer, p53, methylation index (% of loci on chromosomes), RARB methylation	Survival in months	117 patients with 1ry TCC or UCC from one centre	10% CV Kaplan Maier for survival	Average error = 5 months	Interrogate different markers to suggest a predicative combination
[105]	ANN	Bca	Recurrence (classifier)	Age, gender, grade, smoking, previous cancer, p53, methylation index (% of loci on chromosomes), RARB methylation	Recurrence or not	117 patients with 1ry TCC or UCC from one centre	10% cross validation ROC, LR	Ac 89–90%, Se 81–87%, Sp 95–100%	
[105]	ANN	Bca	Survival predictor	Age, gender, grade, smoking, previous cancer, p53, methylation index (% of loci on chromosomes), RARB methylation	Survival in months	117 patients with 1ry TCC or UCC from one centre	10% CV Kaplan Maier for survival	Average error = 9 months	p for comparison ANN and FNM calculated
[128]	ANN	CaP	Diagnosis of cancer in PSA 1–4 4–10	Age, tPSA, %fPSA, TPV, DRE, -5pro PSA, -7, pro PSA	Risk of cancer	2 centre PSA 1–10 and TRUS 6–12 cores, 898 patients	ROC, Spearman correlation co efficient LOO	AUC 84%	Pro PSA improved detection rate in 1–4 and improved %fPSA performance in 4–10 group
[129]	ANN	CaP	Early CaP diagnosis	Age, tPSA, %fPSA, hK11, hK11/tPSA, hK11/%tPSA	Cancer or benign	357 with histologically proven cancer or BPE	ROC Se, Sp test set 206 with histologically proven cancer or BPE	AUC 0.84, Se 90%, Sp 52%	Sensitivity analysis of these variables to demonstrate their impact on AUC
[130]	ANN	CaP	Early CaP diagnosis	Age, tPSA, %f PSA, TPV, DRE (PSA done by five different assays)	Risk of cancer	585 patients with suspected cancer PSA 0.49–27	ROC AUC 25% random set 195 patients and LOO	AUC 0.91 (mean value)	Authors suggests developing PSA assay specific ANN to optimise function
[131]	ANN	CaP	Prostate cancer early diagnosis	Age, BMI, tPSA, fPSA, TPV, PSAD, smoking, systolic-diastolic pressure, pulse, Gl	Cancer or benign	300 patients’ data with suspected cancer from one centre	10- folds CV ROC Se, Sp	Ac 79%, Se 81%, Sp 78%	
[131]	SVM	CaP	Prostate cancer early diagnosis	Age, BMI, tPSA, fPSA, TPV, PSAD, smoking, systolic-diastolic pressure, pulse, Gl	Cancer or benign	300 patients’ data with suspected cancer from one centre	10- folds CV ROC Se, Sp	Ac 81%, Se 84%, Sp 75%	Smoking is a significant classifier but not BMI
[132]	ANN	CaP	Diagnosis	Age, tPSA, %f PSA, DRE, TPV	Risk of cancer	PSA2-20 393proscpective data	ROC AUC LOO	AUC 0.75, Se 90%, Sp 37%	Demonstrate the impact of different data cohorts on ANN performance
[133]	FNM ANN	Bca	Gene micro array to predict UCC progression	200 genes reduced from 2800 by Pearson correlation	Cancer progression to muscle invasive or metastatic	66 tumours from 34 patients in one centre	COX multivariate analysis 10 folds CV	11 new gene signatures	200 gene micro array reduced to 11 gene signatures
[134]	ANN	U Dyn	Urodynamic interpretation	Age, BMI, menopause, sexual activity, UTI, number of vaginal deliveries, surgery,	U Dyn diagnosis	802 data from single centre POP with symptoms and UDS performed	ROC and compare to multi linear regression CV 20%	AUC 80% (Average)	ANN cannot replace Urodynamic
[135]	ANN	Fert	Seminal profile from questionnaire about life habits and health status	Age, BMI, demographic, medical history facts, smoking, alcohol, life style and cloth	Seminal profile	100 volunteers one centre study	ROC AUC Se, 10 Folds cross validation	Se 73–94%, Sp 25–45%, PPV 79–92%, NPV 7.4–54%	Comparison of different AI classifiers with same variables
[135]	SVM	Fert	Seminal profile from questionnaire about life habits and health status	Age, BMI, demographic, medical history facts, smoking, alcohol, life style and cloth	Seminal profile	100 volunteers one centre study	ROC AUC Se, tenfold CV	Se 74_99%, Sp 12–21%, PPV 75–91%, NPV 4–86%	
[135]	DT	Fert	Seminal profile from questionnaire about life habits and health status	Age, BMI, demographic, medical history facts, smoking, alcohol, life style and cloth	Seminal profile	100 volunteers one centre study	ROC AUC Se, tenfold CV	Se 72–96%, Sp 12–41%, PPV 77–90%, NPV 4–48%	
[136]	ANN	Fert	Seminal profile from questionnaire about life habits and health status	Age, season, childhood disease, surgery, trauma, smoking, alcohol, hours sitting ANNA1	Sperm concentration	100 volunteers one centre study	ROC AUC Se, Sp 10 Folds CV	Se 95%, Sp 50%, PPV 93%, NPV 60%	
[136]	ANN	Fert	Seminal profile from questionnaire about life habits and health status	Age, BMI, marital status, vaccines, siblings, allergy, baths, hours of sleep ANNA2	Sperm motility	100 volunteers one centre study	ROC AUC Se, Sp	Se 89%, Sp 44%, PPV 89%, NPV 44%	
[137]	ANN	CaP	Statistical evaluation of PSA INDEX	Age, TPV, DRE, tPSA, %fPSA	Risk of Cancer	1362 from multiple centres with suspected CaP and PSA 1.6–8.0	ROC AUC and comparison to other markers	AUC 0.7—0.74	
[137]	ANN	CaP	Statistical evaluation of PSA INDEX	Age, TPV, DRE, tPSA, %fPSA, %p2PSA	Risk of Cancer	1362 from multiple centres with suspected CaP and PSA 1.6–8.0	ROC AUC and comparison to other markers	AUC 0.73—0.79	
[137]	ANN	CaP	Statistical evaluation of PSA INDEX	Age, TPV, DRE, tPSA, %fPSA, %fPSA prostate health index (p2PSA / fPSA X square root tPSA)	Risk of Cancer	1362 from multiple centres with suspected CaP and PSA 1.6–8.0	ROC AUC and comparison to other markers	AUC 0.73- 0.8	Prostate Health index improved ANN performance
[112]	ANN	Bca	Survival post cystectomy	Age, gender, albumin, surgical approach, tumour stage, follow up period, type of diversion	5 years survival	117 patients with post cystectomy from one centre	ROC, Se, Sp Ac, 10 Folds cross validation	Ac 72–80%	Comparison of 7 different machine learning RELM and ELM had best performance
[138]	ANN	CaP	+ ve lymph nodes to the total number of lymph nodes in predicting BCF	Age, tPSA, Clinical stage, Gl, seminal vesicle invasion, number of positive lymph nodes and laterality of lymph node involvement	BCF	124 cases with lymph node dissection	hazard ration for each variable	LND, Gl, and stage were identified as independent prognostic	LND is more prognostic than their number
[139]	BN	BPE	Correlation between symptoms, decision and outcome of surgery	Age, Qmax, PVR, PSA, TPV, TZV, BOO on UDS, and IPSS scores (stratified)	surgical decision-BN model, the outcome of surgery	1108 cases from one centre	ROC AUC and correlation coefficient	AUC 0.8 TZV (R = 0.396, P < 0.001), treating physician (R = 0.340, P < 0.001) and BOO on UDS (R = 0.300, P < 0.001)	TPV, physician, BOO on UDS, and the IPSS item of intermittency were factors that directly influenced Decision-making in physicians treating patients with LUTS/BPE
[140]	ANN	CaP	Progression biomarkers	Gene microarray	Cancer progression and DSS	192 tissue histology results	MSE for each variable, then Kaplan Meyer and Pearson’s × 2-tests	10 gene microarrays identified by ANN	Ki67 and DLX2, appear to predict CaP specific survival and metastasis
[141]	ANN	VUR	Renal ultrasound to predict voiding cystourethrogram (VCUG)	Renal ultrasound findings	abnormal VCUG	2259 cases post UTI and had VCUG	ROC AUC	Se 64.2%, Sp 59.6%, PPV 61.6%, NPV 62.2%, AUC 0.6852	Renal ultrasound is a poor screening test for VCUG-identified abnormalities

In this application, the system modifies their machine learning ability to identify the significant variables from the data in terms of their correlation to a specified outcome. This can save time, effort and cost specially when applied on gene microarrays

##### Domains

*Prostate cancer* was a common domain in this application with a total of 15 systems analysing predictive factors for diagnosis of cancer, response to treatment and quality of life with prostatic disease. One of the hot topics in Urological cancer is discovering alternative CaP diagnostic markers since serum PSA is not sensitive for distinguishing benign from malignant disease. Stephan et al. investigated the diagnostic value of three markers in this domain: Macrophage inhibitory cytokine-1, macrophage inhibitory factor and human kallikrein 11 [108]. These were used as variables (nodes) in ANN models and compared their accuracy to the linear regression of %fPSA. They have reported that only the ANN model including all three variables was more accurate (AUC 91%, Se 90%, Sp 80%) than all other models proving his hypothesis that they are only relevant as when combined.

Similarly, another study estimated the predictive values of serum PSA precursors (-5, -7 proPSA) in diagnosing prostate cancer using and comparing the accuracy to %fPSA [107]. The -5, -7 pro PSA were only significant in the cohort with PSA between 4 and10 µg/l and did not improve the predictive accuracy when added to the %fPSA. The same author tested this hypothesis on another free PSA precursor (-2 proPSA) by developing ANN with the %p2PSA (-2 ProPSA: fPSA) among other disease variables, which have improved the system accuracy (AUC 85% from 75%) [120].

Three systems evaluated the presence of bcl-2 and p53 (tumor suppressor genes) as a predictive variable for response to prostate cancer treatment [76, 77]. Their combination was reported to be significant (Ac 85%, *p* < 0.00001) in [77] but [76] found that only bcl-2 is relevant in the other two models (accuracy 63–68%).

*Bladder cancer* diagnosis and disease progression was the second most common domain with 13 systems. Kolasa et al. [110] have modeled an ANN with three novel urine markers: urine levels of nuclear matrix protein-22, monocyte chemoattractant protein-1 and urinary intercellular adhesion molecule-1, to predict the diagnosis of bladder cancer and it succeeded in predicting all cancer free patients when the three variables were used as a group. Catto.et al. [119] developed two AI models (ANN & FNM) performing microarray analysis on genes associated with bladder cancer progression. Their models narrowed down these genes from 200 to 11 progression-associated genes out of 200 ([OR] 0.70; 95% [CI] 0.56–0.87), which were found to be more accurate than the regression analysis when compared to the specimen immunohistology results.

Kolasa et al. [110] model predicting the pre-histology diagnosis of malignancy based on urine level of novel tumour markers. Their ANN was found to be more accurate (Se 100%, Sp 75.7%) than haematuria diagnosed on urine dipstick (Se 92.6%, Sp 51.8%) and atypical urine cytology (Se 66.7%, Sp 81%).

*ESWL of renal stones* was the research domain of [30, 69], where they aimed at identifying significant variables correlated to the treatment outcome (stone free) and developing a predictive model. Chiu et al. [69] model did not recognise residual fragments following ESWL as a significant risk for triggering further stone growth and [30] identified these factor: positive BMI, infundibular width (IW) 5 mm, infundibular ureteropelvic angle 45% or more (IUPA), to be all predictive of lower pole stone breaking and clearance.

*Benign prostatic hyperplasia* was modelled in a system [114] to link the disease specific clinical and radiological factors with the disease progression in patients with mild disease (IPSS < 7) and not receiving any treatment. His ANN identified: obstructive symptoms (Oss), PSA of more than 1.5 ng/ml and transitional zone volume of more than 25 cm^3^_,_ to be correlated to disease progression and can accurately predict 78% of the cohort who will need further treatment.

*Urinary dysfunction* diagnosis accuracy by clinical symptoms was compared to urodynamic findings in female patients with pelvic organ prolapse by [115] and both the linear regression and ANN models could not establish relation between the symptoms and urodynamic based diagnosis hence dismissing the hypothesis of only relying on clinical symptoms to reach an accurate diagnosis and replace the need for urodynamics study.

*Hypogonadism* (Hgon) was represented in [133] system where the diagnosis was made based on patient’s age, erectile dysfunction and depression with AUC of 70% (p < 0.01).

#### Image analysis

This one of the advancing applications of AI in medicine where the system either analyse the variables in the reported medical images as data input or identifies these variables through a separate image analyser without the need for expert to report the scan or images. The first category was included among other systems mentioned above as in the diagnosis prediction domain where [47] included different variables from TRUS in the system input to predict CaP diagnosis. In this domain, we focused on the other group where the images are presented to the machine in the form raw data translated by the image analyser and the system will then apply their machine learning to identify the cause effect pattern (Table 8).

**Table 8:** Image analysis

Art	Mdl	Dom	Subdomain	Variables	Output	System training	Validation	Statistical outcome
[142]	ANN	CaP	Radiotherapy dose planning	Patient prostate contour points (anterior, posterior and 5 lateral)	Anterior, posterior and lateral beam	12–68 patients record of radiotherapy treatment planning	Average asymmetry of ANN and acceptance by dosimetrists Small field prostate (n = 133) and for large field prostate (n = 64)	Average asymmetry of ANN 0.20% and acceptance by dosimetrists was 96% (small field prostate) and 88% for large field prostate
[143]	ANN	CaP	Diagnosis of localised disease from TRUS	Pixel distribution and grey levels of the TRUS images	Benign, malignant with Gleason grading	53 images of benign and malignant sample images from 5 patients	Compare to histology results of 500 pictures from 61 patients post RRP for localised disease in one centre	Sp 99%, Se 83%, true positive for isoechoic is 97%
[144]	ANN LDA	CaP	progression post RPP	Prostate volume, PSA, Pathology morphometric variables LDA	Progression or no	Progression t2n0 post RRP, 228 patients from one centre	ROC, Sp, Se, LOO 39 cases	Ac 70%, Se 55%, Sp 85%
[144]	ANN LVQ	CaP	progression post RPP	Prostate volume, PSA, Pathology morphometric variables	Progression or no	Progression t2n0 post RRP, 228 patients from one centre	ROC, Sp, Se, LOO 39 cases	Ac 90%, Se 95%, Sp 85%,
[144]	ANN LVQPAK	CaP	progression post RPP	Prostate volume, PSA, Pathology morphometric variables	Progression or no	Progression t2n0 post RRP, 228 patients from one centre	ROC, Sp, Se, LOO 39 cases	Ac 83%, Se 85%, Sp 80%
[144]	ANN MLFF-bp	CaP	progression post RPP	Prostate volume, PSA, Pathology morphometric variables	Progression or no	Progression t2n0 post RRP, 228 patients from one centre	ROC, Sp, Se, LOO 39 cases	Ac 76%, Se 73%, Sp 80%
[145]	kNN	CaP	TRUS cancer image analysis	Image pixels segmented by tissue descriptor (spatial grey level dependence)	Predict cancer	Images of 202 patients with suspected CaP at one centre	87 randomly selected patients Comparison to other classifiers and ROC	AUC 0.6
[146]	ANN	CaP	TRUS Image segmentation	Pixel’s colour values from TRUS images	TRUS image segmentation	212 CaP TRUS data	Overlap measure (compared to expert segmented boundary) on 10 random images	81% mean overlap measurement
[147]	ANN	CaP	MRI cancer diagnosis	256 MRSI spectra (resonance intensities at given PPM)	Cancer or benign	5308 voxels of 18 patients with CaP in a retrospective study	15% validation ROC Se, Sp	AUC 0.95, Se 50%, Sp 99%
[147]	ANN	CaP	MRI cancer diagnosis	256 MRSI spectra (resonance intensities at given PPM), peripheral and transition zone, periurethral and outside region	Cancer or benign	5308 voxels of 18 patients with CaP in a retrospective study	15% CV validation ROC Se, Sp	AUC 0.97, Se 62%, Sp 99%
[148]	SVM	CaP	Diagnosis of cancer from pMRI images	Image segmentation then clustering voxels	Cancer or benign	16 pMRI images with CaP	Correlation coefficients of voxel parameters	Mean accuracy of 84%
[149]	ANN	Bca	Image histology analysis	Image histology analysis (measurements of the segmentation of nuclei and other features)	Benign and malignant	141 randomly chosen cell images (30%)	329 cell images (70%) ROC, Sp, Se	Sp 100%, Se 82%, PPV 96%, NPV 80%, Ac 88%
[150]	FCM	Bca	Diagnosis of tumour	Bladder wall segmentation and tumour region extraction To detect bladder abnormalities, four volume-based morphological features: bent rate, shape index, wall thickness, and bent rate difference between the inner and outer surfaces	Bladder neoplasm	16 Bladder tumour MRI images	Overlap Ratio (OR)	OR 86.3%
[151]	ANN	Bca	Transitional cell cytology analysis	Cytology image analysis and pixel variations as variables	Cancer or benign	16 cytology images	comparison to experts, × 2 test	75% concordance with the experts
[152]	ANN	Nlt	Spectroscopy stone analysis	Absorbance infra-red spectrum of 91 wave lengths	Stone composition	160 and 57 stone mixtures	Predictive accuracy, root mean square error on 36 independent stone samples	Overall good predictive value

Expert Systems in this application analysed images from histology and radiological scans to learn patterns that are correlated to a specific diagnosis. They have proven to be effective in this domain and they facilitated diagnosis of cancer and even delivering radiotherapy dosage

##### Domains

*Prostate cancer* image analysis was modelled in 10 systems to enhance diagnostic accuracy as in [126] and disease progression prediction as in [128]. The first system represented each TRUS image pixel as one variable or neuron in a pulse coupled neural network and trained their system with 212 prostate cancer images to segment prostate gland boundary with an average overlap accuracy (overlap measure = difference between PCNN boundary and the expert) of 81% for ten images [126].

The other 4 systems analysed histological images of a cohort of patients post RP with clinically localised CaP to predict the disease progression. The histological images were given coloured coding and analysed by the system that used variables as % of epithelial cell and glandular Lumina to identify the high risk group for disease recurrence with an accuracy reaching 90% [128].

*LUT* disease urine cytology images were analysed by 2 models in [123], which identified all patients with benign disease with an overall accuracy of 97%.

*Nephrolithiasis* stone biochemistry analysis can be achieved through an expert analysis of infrared spectroscopy which was simulated by [124] where the infrared spectra wavelength numbers were modelled as input variables and the system prediction accuracy of the expert analysed stone specimen had a root square mean error of 3.471.

### Qualitative analysis

The same articles were considered for the qualitative analysis against the four stages (validation, verification, evaluations and credibility) reported in Okeefe industrial survey [8] and Benbasat article [9]. The completion of the four stages examined in this qualitative analysis was demonstrated by none of the included systems. There is a possibility that some of these missing stages has been performed but not published in the scientific literature.

Validation was performed by almost all the systems (166 out 169) with varying degree of study strength, bias, and limitations (Table 9). Most of the data driven systems (ANN, SVM, BN, kNN and FNM) were validated by the ROC and AUC by having a training and validation set or cross validation or applying the leave one out technique. Samli et al. enhanced the validity of their system by estimating the kappa statistics with the ROC [134].

**Table 9:** Qualitative assessment of urological Expert Systems

Art	Mdl	Validation methods	Credibility	Evaluation	Validation	Verification	Strength and bias
[27]	RBR	Patients' evaluation	No	Yes	Yes	No	Only qualitative evaluation
[18]	RBR	Blinded comparison against 4 experts with independent experts rating and 3 centres RCT pilot trial	Yes	Yes	Yes	No	Consideration of system evaluation with real time testing but small number
[21]	FRB	Improve practitioner accuracy	No	No	No	No	Insufficient info on development and validation
[15]	RBR	RCT reliability and validity by experts’ reviews	Yes	Yes	Yes	No	Small number in the study and short duration of follow up
[95]	ANN	ROC, Sp, Se	No	No	Yes	No	Small number for validation
[63]	FSS	ROC, Sp, Se	No	No	Yes	No	2 methods for validation, compared to experts and data
[143]	ANN	Compare to histology results	No	No	Yes	No	No comparison to human to demonstrate usability, no p value or CI
[103]	FNM	ROC, LR, RMS	No	No	Yes	No	p value calculated to compare all models
[103]	ANN	ROC, LR, RMS	No	No	Yes	No	p value calculated to compare all models, the effect of combining HK p53 with other variables
[102]	ANN	ROC, Sp, Se	No	No	Yes	No	No p value
[76]	ANN	Correlation co-efficient	No	No	Yes	No	Correlation co-efficient between expert and system? Kappa more accurate
[40]	FRB	Not published	No	No	No	No	Not validated
[68]	ANN	AUC ROC	No	No	Yes	No	p value calculated vs LR
[19]	RBR	Feedback from patients with no control group	No	Yes	No	No	No validation but user (patient evaluation)
[29]	FRB	Comparison to experts and non-experts	No	No	Yes	No	Expert as gold standard
[25]	RBR	PPV 62%, NPV 100% Se 100% Sp 33%	No	No	Yes	No	Small number, low specificity
[55]	ANN	ROC AUC then compare with LR, kappa stats	No	No	Yes	No	Multimodal of validation
[99]	ANN	ROC, Sp, Se	No	No	Yes	No	Not long term follows up
[43]	ANN	ROC (0.74 and 0.86)	No	No	Yes	No	TRUS finding from expert panel, human as gold standard
[105]	FNM	ROC, LR	No	No	Yes	No	p value calculated to compare all models
[105]	ANN	Kaplan Maier for survival	No	No	Yes	No	p for comparison ANN and FNM calculated
[145]	kNN	Comparison to other classifiers and ROC	No	Yes	Yes	No	Evaluated the usability of the product and was found to have less than significant effect
[129]	ANN	ROC Se, Sp	No	No	Yes	No	Sensitivity analysis of input variables
[22]	ANN	ROC 0.7, accuracy 79%	No	No	Yes	No	Compare to experts without accounting for human error
[85]	FRB	ROC Se, Sp	No	No	Yes	No	No user evaluation
[24]	FRB	Ac 0.76, Se 0.79, Sp 0.75	No	No	Yes	No	Expert as gold standard
[109]	ANN	ROC Compare to LR	No	No	Yes	No	CI calculated
[12]	FRB	Ac 0.93, Se 0.97, Sp 0.99	No	No	Yes	No	Expert as gold standard
[110]	ANN	Prediction error percent	No	No	Yes	No	Experimental results
[48]	SVM	ROC AUC	No	No	Yes	No	P value calculated to compare all models
[146]	ANN	Overlap measure (segmented by experts)	No	No	Yes	No	Expert as gold standard
[23]	ANN	Ac 0.84, Se 0.93, Sp 0.33	No	No	Yes	No	Experts verified data no account for human error
[30]	FNM	Accuracy 86.8%	No	No	Yes	No	Guidelines as gold standard
[20]	RBR	Evaluation by experts, 95 retrospective	No	No	Yes	No	Expert as gold standard, qualitative evaluation
[26]	HYB FUZZY ONT	Kappa vs experts, k = 0.89	No	No	Yes	No	Kappa limitation prospective, randomisation,
[16]	RBR	Se 0.95, Sp 0.72, Bayesian analysis S&S, usability of system by Likert scale (Cronbach’s alpha 0.9)	Yes	Yes	Yes	No	Full system evaluation but nurse as gold standard, no attempts to eliminate error
[91]	ANN	ROC AUC compare with Partin nomogram and LR	No	No	Yes	No	No correlation with user
[17]	FNM	Kappa vs experts, Se 0.95, Sp 0.92	No	No	Yes	No	Human expert as gold standard and no qualitative evaluation (weight of error)
[60]	ANN	Ac 60% (testing) 75% (training)	No	No	Yes	No	Compare to gold standard, Urodynamic
[117]	ANN	PPV 100%	No	No	Yes	No	No calculation of NPP and overall accuracy
[32]	FNM	Correlation coefficient = 0.99	No	No	Yes	No	Small number of cases for validation
[150]	FCM	OR 86.3%	No	No	Yes	No	Comparison with experts as gold standard than mapping to histology
[141]	ANN	ROC, Se 64.2%, Sp 59.6%, PPV 61.6%, NPV 62.2%, AUC 0.6852	No	No	Yes	No	Similar to urodynamic as research tool
[54]	FRB	None	No	No	Yes	No	No validation

All systems’ development was qualitatively assessed against the common industrial steps in the development pathway described by Okeefe and Benbasat. With exception of the system validation, the rest of the cycle was defective with no explanation. The validation had variable degree of strength with common application of the receiver operator characteristic for estimating the area under the curve for data driven systems

Evaluation was only performed by a small fraction of these systems (n = 6). Their evaluation was aiming at the user or the expert but rarely both. There is no evidence to support that these were performed at early stages to determine the substantiality of the system to the user.

System credibility and verification were never performed. It would be implied that the verification was performed to an extent but not reported as it is a technical part of the development.

‘System development limitation and bias evaluation’ demonstrated an overall acceptable validation methodology with valid statistical analysis. However, a few observed limitations (Table 9) were reported with the common encounter being the consideration human opinion as a gold standard (n = 9). For instance, the gold standard in diagnosing prostate cancer is tissue biopsy confirmation. The interpretation of the expert clinical diagnosis as the gold standard reference can lead to statistical errors and invalidate the study.

## Discussion

**Fig. 4:**
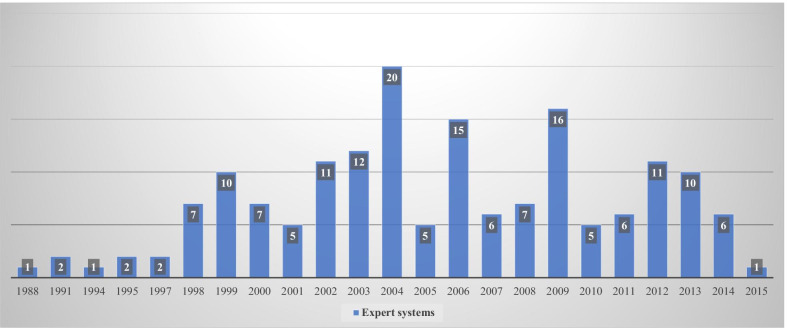
Expert System (ES) analysis by year of publication showing an upward trend and increase in number of publications. Systems were included according to the keywords for expert system models and applied in urological domains

Expert Systems are widely available in Urological domains, with a large range of models, applications, domains, and target users including patients, students, non-experts, experts, and researchers. The number of published systems has risen over the years but with a consistent lack of publications reporting their real time testing or healthcare implementation (Fig. 4).

There is an increasing interest in analysing this gap which is reflected from the scope of AI historic review articles which aimed to only familiarise the readers with ES existence and application [33, 125]. In fact, the majority had a relatively narrow scope on the evolution and application of one ES models (artificial neural network) in prostate cancer diagnosis. Recently, similar to our research, there has been more interest in AI validation, and lack of uptake despite the faith in their ability. Therefore, in this study we quantified ES progression and applications in Urology while examining their developmental life cycle.

It was evident that CaP was the commonest domain in almost all applications contributing with more than two thirds of the systems (91 systems in total). Different aspects of this domain have been simulated by these systems to include diagnosis, therapeutics, predictions of disease progression or treatment outcome, researching variables and medical images analysis. Most of these systems were simulating urologist cognitive function with little guidance on their benefits and how they can be implemented to improve cancer decision making.

In industry, this is usually performed before the system development by evaluating the system usability from the user perspective. This part has lacked or not been acknowledged in the published studies and is possibly a core reason for the lack of their integration in urological health care. Furthermore, none of these systems has been a subject to live testing in a well-designed study to prove its efficacy over standard tools or in the clinical context to prove its validity to justify their complex structure to AI novice health care professionals. The qualitative analysis demonstrated that validation is the only stage of the development cycle to be applied by most of the systems and there is a lack of system evaluation, credibility, and verification. The evaluation can be subdivided into usability (usually by average user), utility and system quality (by experts) [9]. Despite the crucial stage of ES development, there has been a lack of attention in the published articles to integrate it into the development life cycle. This can mean the whole system can fail and also challenge its uptake [8].

An example can be drawn from this review where the majority of the systems focused on CaP diagnosis and treatment. Their implementation would be challenged by the standard decision-making tools of the cancer multidisciplinary team and the ethical concerns of relying on ANN in making such life changing and expensive decision. The utility analysis of those ES would have been essential for tailoring their development for real time applications where they can be more substantial to the user. One example is lack of community-based systems for the initial referral of suspected cancer patients and follow up of stable disease, where NICE have identified a need for such decision support models [152, 153].

There was a wide diversity of modelling in Urological ES with ANN being the most common model in this review. These would bypass the need for direct learning from experts and the exhaustive process of knowledge acquisition, which is a core requirement for knowledge-based systems to attest the whole system progress [55]. However, their analytical hidden layer of nodes “black box phenomenon” has been a subject for wide criticism and rejection from clinicians due to lack of transparency and understanding of its function.

Stephan et al. suggested a statistical solution to identify the variables significance by performing sensitivity analysis [154]. This estimates the variation of the AUC with introduction or elimination of each variable. This can only reflect the significance of each variable but does not explain how the cases are being solved nor quantify this to the user in a standard statistical value. This can be useful in research as they can identify significant variables in a large set data and has been successfully applied in the field of academic urology as in [119] where the system successfully identified the relevant gene signature for bladder cancer progression which saved time and cost of microarray analysis of all suspected genes.

*Holzinger *et al. emphasised on the importance of the explicability of the AI model specially in medicine which is a clear challenge for machine learning due to their complex reasoning [155]. Their study attempted to simplify the explanation by classifying the systems into post-hoc or ante-hoc. In post-hoc, explanations were provided for a specific decision as in model agnostic framework where the black box reasoning can be explained through transparent approximations of the mathematical models and variable [156, 157]. Those are reproduced on demand for a specific problem rather than the whole system which can shed more light on the system function. It is not certain if those can be easily interpreted by the AI novice clinician, but it has provided more explicit models for tackling the black box phenomenon.

Knowledge based systems can be explained by ante hoc models where the whole system reasoning can be represented. Those systems rely on expert knowledge in their development and face the bottle neck phenomenon in their applications. Furthermore, they are not always successful in identifying and mapping multilinear mathematical rules and machine learning is mandatory or at least more efficient [155]. Bologna and Hayashi et al. suggested that machine learning is more successful in complex problem solving with inverse relation between the machine performance, and it is built-in transparency [158].

Another common aspect lacking in these articles was the coupling of their system development methodology with the medical device registration requirements. This is essential as ES often function as standalone software with no human supervision to their calculation. This categorises the system as a medical device with mandatory perquisite to register with the relevant authorities as Medicines & Healthcare products Regulatory Agency in the UK [5].

Cabitza et al. compared AI validation to other medical interventions as drugs and emphasised on considering the “software as a medical device” [159]. Unlike other devices or drugs, AI models in healthcare are unique in being more dynamic which should be reflected in their validation cycle. They also quoted the known term “techno-vigilance” to learn from other medical device validation pathways. They recommended different outlook to validation where it is broken down to statistical (efficacy), relational (usability), pragmatic (effectiveness) and ecological (cost-effectiveness) with available standards for those steps (ISO 5725, ISO 9241 and ISO 14155). The latter is viewed as a novel standard for evaluating the cost benefits of applying specific AI model in healthcare which would require longitudinal modelling of health economics [159]. This was evidently lacking in articles that were included in our review and in fact most of the studies were non-randomised and retrospective.

Similarly, Nagendran et al. systematically analysed studies that compare AI performance to experts in classifying medical imaging into diseased and non-diseased, they concluded that AI performance was non-inferior to human experts with potential for out-performing [160]. Their 10 years review identified from literature 2 randomised clinical trials and 9 prospective non-randomised trials extracted from a total of 10 and 81 studies, respectively. Their review assessed the risk of bias using PROBAST (prediction model risk of bias assessment tool) criteria for non-randomised studies. The tool is designed for identifying the risk of bias by analysing four domains (participant, predictors, outcome, and analysis) [161], which is applicable to systematic review analysing prediction model with a target outcome.

In our study, as there was no unified outcome for the included prediction tools, the scope was on the role of validation rather than the outcome. Therefore, those tools assessing the risk of bias were not utilised due to the wide gaps in the tool checklist between the included articles. Such study design and data heterogeneities were also evident in Nagendran et al. and similar to our study, data synthesis was not possible. This will pose a challenge reinforcing the application of AI models in healthcare due to lack of level 1 evidence which is mandatory in healthcare for accepting a novel intervention.

Finally, the quality of the data analysis was beyond the scope of our systematic review despite being essential for developing quality AI systems. Cabitza et al. examined this gap and focused on the data governance [161]. There has been very limited evidence on data quality appraisal and standards with call for further research and allocation of more resources specially in healthcare where the data are notoriously limited with errors or discordance.

The potential application of AI in urology with focus on its future application has been recently discussed by Eminaga et al. [162]. They have shown an increasing interest in urology research, but with a challenged mechanistic update due to the model complexity and lack of end user understanding of its design and function. Furthermore, they identified discrepancy between AI engineering and clinical application which reflects some lack of communication between both disciplines.

This can be either a consequence or a cause for lack of clinical utility testing, which increases the need for research in this domain to be incorporated in the software development [163]. In fact, it has been recommended to perform the utility test before developing the system to tailor its application [164, 165]. Despite having different methodology to our systematic review, the recommendations were similar with strong emphasis on the lack of utility testing and its impact on AI uptake in healthcare [166–168].

## Conclusion

ES have been advancing in Urology with demonstrated versatility and efficacy. They have suffered from lack of formality in their development, testing and methodology for registration, which has limited their uptake. Future research is recommended in identifying criteria for successful functional domain applications, knowledge engineering and integrating the system development with the registration requirement for their future implementation in the health care systems.

## Data Availability

For data and supporting materials access, please contact authors for data requests.

## Abbreviations

Ac: Accuracy
AI: Artificial intelligence
ANN: Artificial neural networks
AP: Acute prostatitis
Bca: Bladder cancer
BC: Backward chaining
BCF: Biochemical failure
BCG: Bacille Calmette–Guérin
BP: Back propagation neural network
BPD: Benign prostatic disease
BPH: Benign prostatic hyperplasia
CAD: Computer aided diagnosis
CBR: Case based reasoning
CP: Chronic prostatitis
CV: Cross validation
Dom: Domain
DRE: Digital rectal exam
ED: Erectile dysfunction
ES: Expert Systems
FC: Forward chaining
Fert: Fertility
FH: Family history
FLS: Fuzzy logic systems
F-ONT: Fuzzy ontology
FNM: Fuzzy neural modelling
FRB: Fuzzy rule-based systems
FSH: Follicular stimulating hormone level
GA: Genetic algorithm
Gl: Gleason score
Hgon: Hypogonadism
Hk11: Human kallikrein 11
Incont: Incontinence
IS: Information systems
ISS: Irritative symptoms
IT: Information technology
IUPA: Infundibular ureteropelvic angle
IW: Infundibular width
KA: Knowledge acquisition
KMSP: Kaplan Meir Survival Plot
KE: Knowledge engineer
Lap: Laparoscopy
LH: Luteinising hormone level
LOO: Leave one out
LUT: Lower urinary tract
LVQ: Learning vector quanitizer
MIC-1: Macrophage inhibitory cytokine-1
MIF: Macrophage inhibitory factor
MH: Medical history
ML: Machine learning
MHRA: Medicines and Healthcare products Regulatory Agency
Mdl: Model
Nep: Nephrectomy
Nlt: Nephrolithiasis
NICE: National Institute for Health and Care Excellence
Nomo: Nomogram
NPV: Negative predictive value
Nsc: None seminoma testicular cancer
Oss: Obstructive symptoms
Pop: Pelvic organ prolapse
Pca: Prostate cancer
PPV: Positive predictive value
PRL: Prolactin level
PSAd: PSA density
PSAv: PSA velocity
PVR: Post void residual
Qmax: Maximum flow rate
RA: Requirement analysis
RBR: Rule based reasoning
RC: Radical cystectomy
RCC: Renal cell carcinoma
Recur: Recurrence
Res: Response
ROC: Receiver operating characteristic
RP: Radical prostatectomy
Sc: Single centre
Se: Sensitivity
SPC: Stable prostate cancer
Sp: Specificity
tPSA: Total PSA
TPV: Total prostatic volume
TRUS: Trans rectal ultrasound scan
TT: Total Testosterone
TZD: Transitional zone PSA density
TZV: Transitional zone volume
U Dyn: Urodynamic study
U Dys: Urinary dysfunction
UTI: Urinary tract infection
V&V: Verification and validation
VU rflx: Vesico-ureteric reflux
%fPSA: Percentage free/total PSA
%p2PSA: Percentage p2PSA/fPSA
p2PSA: -2 ProPSA
U incont: Urinary incontinence
